# Light availability, not soil texture, correlates with shrub community structure in mesic forest gaps

**DOI:** 10.1371/journal.pone.0361488

**Published:** 2026-10-29

**Authors:** Sam W. Anderson, Katherine A. McCulloh

**Affiliations:** Department of Biology, University of Wisconsin, Madison, Wisconsin, United States of America; University of Udine: Universita degli Studi di Udine, ITALY

## Abstract

Woody shrubs are important components of temperate forest understories, contributing to forest structure, diversity, and succession. However, the extent to which variation in canopy openness and soil texture influence shrub occurrence, diversity, and composition remains unclear. Because woody tissues require comparatively high light availability and sandy soils may favor shrubs over trees, we hypothesized that shrub richness and cover would increase in canopy gaps and sandier microsites. To address this, we quantified soil texture, canopy gap structure, and recorded shrub abundance in twenty randomly stratified plots within sixteen different mesic forests in Wisconsin, USA. Using a mixed-effects modeling approach, we determined that shrub richness, Shannon diversity, and coverage increased significantly in more open canopy gaps, while soil sand content had no significant impact on these metrics. Canopy gaps also had a significant effect on the composition of shrub communities at the plot level. At the stand level, neither sandier nor more open-canopied forests had more diverse, dense shrub layers, though more heterogenous canopies had a weak negative correlation with shrub richness. Broadly, shrub abundance and cover increased in more open gaps, though individual species demonstrated significantly different preferences in terms of light availability and soil sand content. Our results demonstrate the importance of local light availability for shrub growth. However, species-specific responses to soil texture and light availability and the modeled importance of unmeasured site-level factors suggest that additional environmental and biotic factors are likely to impact this diverse temperate guild.

## Introduction

Under the dense canopies of temperate hardwood mesic forests in eastern North America, light availability during the growing season presents a substantial physiological constraint for understory flora. To maximize growth and reproductive fitness, understory vegetation must either adapt their physiology and life history to reduced light availability or take advantage of canopy gaps that foster brightly lit microsites [1–3]. Mesic forests are known to overlay the broad range of bedrock and soil types due to past glacial erosion and deposition, but the generally nutrient-rich substrates and water availability fosters light competition amongst mesic forest trees like Sugar Maple (*Acer saccharum*), Yellow Birch (*Betula alleghaniensis*) and Basswood (*Tilia americana*) [4,5]. These conditions also inhibit stand-level forest disturbances such as fire and drought, though prolific logging has resulted in mostly secondary forests [4–9]. In older (>70 years since disturbance) mesic forests, the natural disturbance regime is primarily driven by canopy gap formation due to individual treefall driven by storm disturbance (ex. lightning, wind), resulting in gaps roughly the size of 1–2 tree crowns [10–12]. These gaps and the subsequent mosaic of understory light availability and soil disturbance (i.e., nurse logs, pit-mount microtopography) can have significant impacts on herb, shrub, and tree recruitment and growth [12–16].

Within forest understories, co-occurring guilds of plants like shrubs, tree seedlings, and herbaceous plants all acclimate and adapt to low light levels [3,17,18]. In adapting to low light, species can shift their phenology to capture light prior to seasonal canopy closure [3], alter their physiology and anatomy to reduce minimum light requirements for net positive carbon gain (i.e., whole-plant compensation point) [2], or rely on irregularly occurring high-light microsites that occur throughout gap-phase succession [1,2,19–21]. Within the understory flora, the specific life history of shrubs makes their study in relation to light availability particularly informative from an ecological, physiological, and evolutionary perspective. Unlike herbs, shrubs produce secondary woody growth that persists for multiple years [22,23], requiring a higher whole-plant compensation point to meet the energetic needs to grow and maintain tissues, reproduce, and store energy for potential damage, disease, or herbivory [2]. Additionally, woody growth allows for plants to grow taller and root deeper than competing herbaceous vegetation, implying the need for an increased return on investment in terms of light interception and carbon fixation. Though both trees and shrubs are woody perennials, many trees bide their time in the understory until they reach the canopy where the greater light intensity provides the energy for reproduction [18,24]. In contrast, shrubs must complete their entire life cycle within the understory. Since understory shrubs incur the inherent construction costs of woody growth without the obvious gains in terms of increasing light interception due to increased height, the structure of understory shrub communities and species’ preferences for light availability is important for our ecological and functional understanding of shade tolerance in woody plants.

Beyond light, other environmental and ecological factors potentially impact understory shrub communities. Temperate forests can include steep terrain, pockets of soil texture altered by tree fall and nurse logs, and saturated substrates near wetlands, ephemeral ponds, or riparian areas [25]. This heterogeneity in light, soil texture, and water availability fosters various microsites, providing potential for niche partitioning amongst understory flora [26,27]. In particular, soil sand (particles 0.05 mm-2.0 mm in size) content serves as a common proxy for nutrient availability because of faster water infiltration rates and nutrient leeching in sandy soils often result in lower water and nutrient availability [28,29]. These conditions favor a more rich shrub community than one dominated by trees, whose allocation to shoot biomass is favored in conditions with ample nutrient and water availability [2,30]. Microsite heterogeneity and niche partitioning become particularly relevant when considering how shrub habitats and distribution are often characterized as clustered and modal [31,32]. Historical studies of Wisconsin (WI) forests have shown that soil texture, moisture availability, soil fertility, and light exposure are important driving factors in overstory composition and succession, but how these gradients directly relate to understory shrub community composition remains comparatively understudied [33,34]. Shrubs can significantly impact tree seedling growth and recruitment, contribute to structural heterogeneity and habitat, provide year-round food resources, and are often important pollinator resources early in the spring [14,35,36]. They also impose an additional layer of light interception for any underlying herbs, significantly impacting herb understory diversity, evenness, and composition throughout forest succession [37–40]. With shrub species comprising ~60% of the woody species within Wisconsin’s flora, a better quantified understanding of shrub composition serves to both improve our understanding of forest structure and inform our approach to management of forest services, habitat, and biodiversity [41–43].

We surveyed the understories of WI upland forest systems to directly characterize the composition of temperate shrub communities and the microsite specificity of individual species in relation to local light and soil conditions. Given that WI upland forests face increasing mesophication (i.e., more densely shaded forests composed of shade tolerant species), edge effects (ex. logging, housing/urban development), and biological homogenization (declining beta diversity between forest stands), the light and soil microsites explaining shrub presence and abundance within temperate forest communities are of particular interest [44–48]. Characterizing species-level models in relation to environmental variables also informs land managers tasked with prioritizing specific species, forest structure, or ecosystem services [42].

To address this knowledge gap, we used a randomly stratified surveying method to measure shrub presence, coverage, and abundance in twenty plots within sixteen mesic forests in addition to plot-level canopy structure and soil texture. We hypothesized that shrub richness, diversity, and coverage would be positively correlated with both soil sand content and light availability. At the site level, we would expect sites with more heterogenous canopies with a range of soil textures to support a more diverse, dense shrub layer. At the species level, we hypothesized a higher density of shrubs in more open, sandy microsites, with individual species varying in their affinity for light availability or loamy soils.

## Methods

### Field data collection

Forests sites were identified using previous characterizations as mesic or dry mesic through the Wisconsin State Natural Areas program, based on the existence of a canopy dominated by sugar maple (*Acer saccharum*), yellow birch (*Betula alleghaniensis*), and American basswood (*Tilia americana*) [49,50]. To account for potential regional differences in climate and substrate, mesic forests were evenly separated between northern and southern WI (S1 Fig and S1 Table) [50]. From potential sites, State Natural Areas and areas stewarded by the University of Wisconsin-Madison Arboretum that were greater than 5.0 ha with at least seventy years since any previous major disturbance (i.e., blowdown, fire, logging) were selected, with research permits granted by the Wisconsin DNR, U.S. Forest Service, and UW-Arboretum for respective properties. Site selection (n = 16) was confirmed with visual assessment, followed by random selection of sampling locations in the field. 50 m^2^ circular plots (n = 20) were randomly stratified along four parallel transects that were separated by 75 m to ensure that quadrats would not overlap, resulting in a total of 320 plots surveyed across sites. In each plot, shrub species presence, estimated coverage (%), and stem counts (abundance) were recorded. If multiple shoots were obviously originating from the same rootstock, they were considered one stem. Visual estimates of species percent cover were binned into cover classes from 0–5%, 5–25%, 25–50%, 50–75%, 75–95%, and 95–100%, and plot-level cover classes were based on the sum of species’ percentages [51]. *Crataegus, Amelanchier*, and *Ribes* were recorded at the genus level due to the absence of diagnostic floral/fruit characteristics. Species accumulation curves constructed for each site confirmed that twenty plots accurately captured site level species richness (S2 Fig).

A ~ 300 g soil sample was taken from the top 15 cm of mineral soil, with three random soil cores in each plot taken to confirm A1 soil horizon depth. Soil texture was quantified using the hydrometer method of the University of Wisconsin Soils and Forage Lab [52]. While soil nutrient analysis can better characterize nutrient availability, soil texture analysis was used as a proxy due to the number of plots surveyed [29]. Canopy structure was quantified using spherical panoramic photographs that were taken using a Google Pixel 4a located 1.25 m above the soil surface at the center of the plot [53]. To minimize direct solar interference, photos were taken in early morning, late afternoon, or on overcast days to avoid direct irradiance. Spherical panoramas were converted to hemispheric photographs and binarized using ImageJ [54]. Binarized hemispheric photographs were verified to confirm that direct sunlight was not altering subsequent measurements. Canopy metrics were quantified using both the R package *HemispheR* (v.1.1.4) and ImageJ add-on *Hemispheric2.0* to corroborate multiple metrics of gap structure [55,56]. Many canopy metrics such as gap fraction displayed a logarithmic distribution and were log-transformed prior to modelling. Given the methods used to place understory plots, canopy structure ranged from near complete coverage to openings that occupied up to 12% of the canopy. These gaps were typically the size of a single tree crown and were formed by dead standing timber, storm damage to the canopy, or entire trees falling.

To test whether species composition is driven by location or broader climatic variables, site level climate data were interpolated from WorldClim data [57] using R package *raster* (v3.6-32) [58]. Among these bioclimatic variables, mean annual temperature (MAT, °C) and Annual Precipitation (MAP, mm) were selected for inclusion in analyses based on *a priori* hypotheses derived from robust vegetative surveys for herbaceous and overstory species in WI forests and variables known to co-vary with latitude in WI [4,59,60].

### Statistical analysis

We used various hierarchal mixed models to directly test the effects of canopy openness and soil texture on community variables (i.e., species richness, diversity, cover class) and species level variables (i.e., presence, abundance, cover class) [61,62]. Biodiversity analyses at the site and plot level were performed in r package *vegan* (v2.6-8) [63]. Diversity models and richness models were constructed using R package *lme4*(1.1–35.5) using Poisson and Gaussian distributions respectively, and plot cover class models were built using ordinal logistic regression in R package *ordinal* [64–66]. For plot level models, log-transformed canopy gap fraction and sand were included as fixed effects, while site was included as a random effect to account for the hierarchal nature of the sampling protocol. Community composition was assessed on plots with at least one species (n = 288) using non-metric multidimensional scaling of shrub abundance data using Bray-Curtis Dissimilarity [67,68]. Environmental variables were vectorized, and the correlation of these environmental variables with NMDS axes was assessed using non-parametric statistical tests (PERMANOVA) to partition community composition-derived distance matrices among sources of variation (*i.e.,* environmental conditions) [69,70].

At the species-level, multilevel models were constructed using species presence/absence at the plot level, followed by presence-only models of abundance (stem counts) and cover class as the response variables [61]. Only species that were found in fifteen or more plots (n = 22) were included in modelling. Shrub presence, abundance, and coverage were modelled using a Bayesian mixed modelling approach using R package *brms* (2.22.0) [71]*.* Hierarchal models were chosen to account for both the nestedness of plots within sites and the simultaneous modelling of multiple species [72]. Presence was modelled using a binomial distribution on all presence/absence observations, while abundance was modelled using a negative binomial distribution, and cover class was modelled using logistic ordinal regression. All models included canopy gap fraction, soil sand content, and a quadratic term for gap fraction as fixed effects to account for the potential nonlinear, unimodal relationship between canopy gap fraction and abundance observed in exploratory data visualizations. Site, plot, and species-level random effects were included in all models to account for differing intercepts and slopes of fixed effects of each species and the hierarchal structure of the sample design.

Bayesian mixed-effects models were fit using weakly informative priors. Models were run with four Markov chain Monte Carlo (MCMC) chains of 4,000 iterations each, including a 1,000-iteration warm-up period. Effects were considered meaningful when their 95% credible interval did not overlap zero. Predicted responses were visualized using *sjPlot* (2.9.0) [73]. Model performance was evaluated using posterior predictive checks, and the importance of site- and plot- level random effects was assessed by comparing models with and without these terms using leave-one-out cross-validation (PSIS-LOO), with lower values indicating better predictive performance [72,74].

## Results

### Community level analysis

Forty-two taxa were observed throughout surveys, with twenty-two taxa found in fifteen or more plots. Across both southern and northern WI, shrub species richness (*z* = 2.323*, p* = 0.0202), Shannon diversity (*z* = 2.124, *p* = 0.0245), and coverage (*z* = 2.643, *p* = 0.00823) were positively correlated with gap fraction (Figs 1 and 2 and S1 Table). While significant, fixed effects only explained 4.6%, 5.5%, and 4.3% of the variation in species richness, diversity, and coverage respectively (S2 Table). Soil sand content showed no significant impact on shrub richness or Shannon diversity (S2 Table). At the plot level, there is no significant difference in richness between southern and northern forests, with most of the variation occurring at the site and plot level (Fig 1 and S2 Table). However, cover class was significantly higher in southern forests than those in the north (*z* = 467.710*, p*=<0.001, S2 Table).

**Fig 1:**
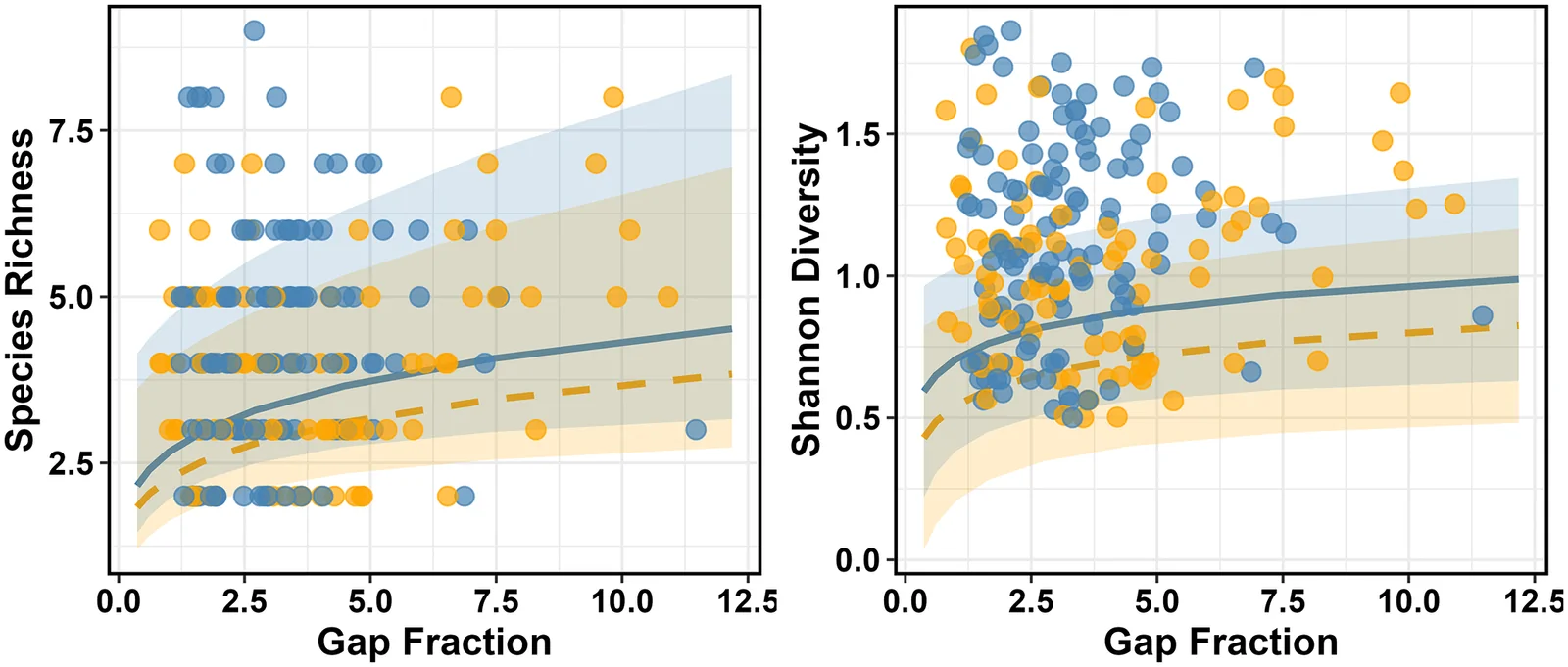
Modelled relationships between diversity metrics and canopy gap fraction. Modelled significant relationships between gap fraction and a) species richness and b) Shannon diversity. Each dot in a) and b) represent a plot, with color denoting plots in northern (orange) and southern (blue) forests, though regional differences in diversity metrics were not significant.

**Fig 2:**
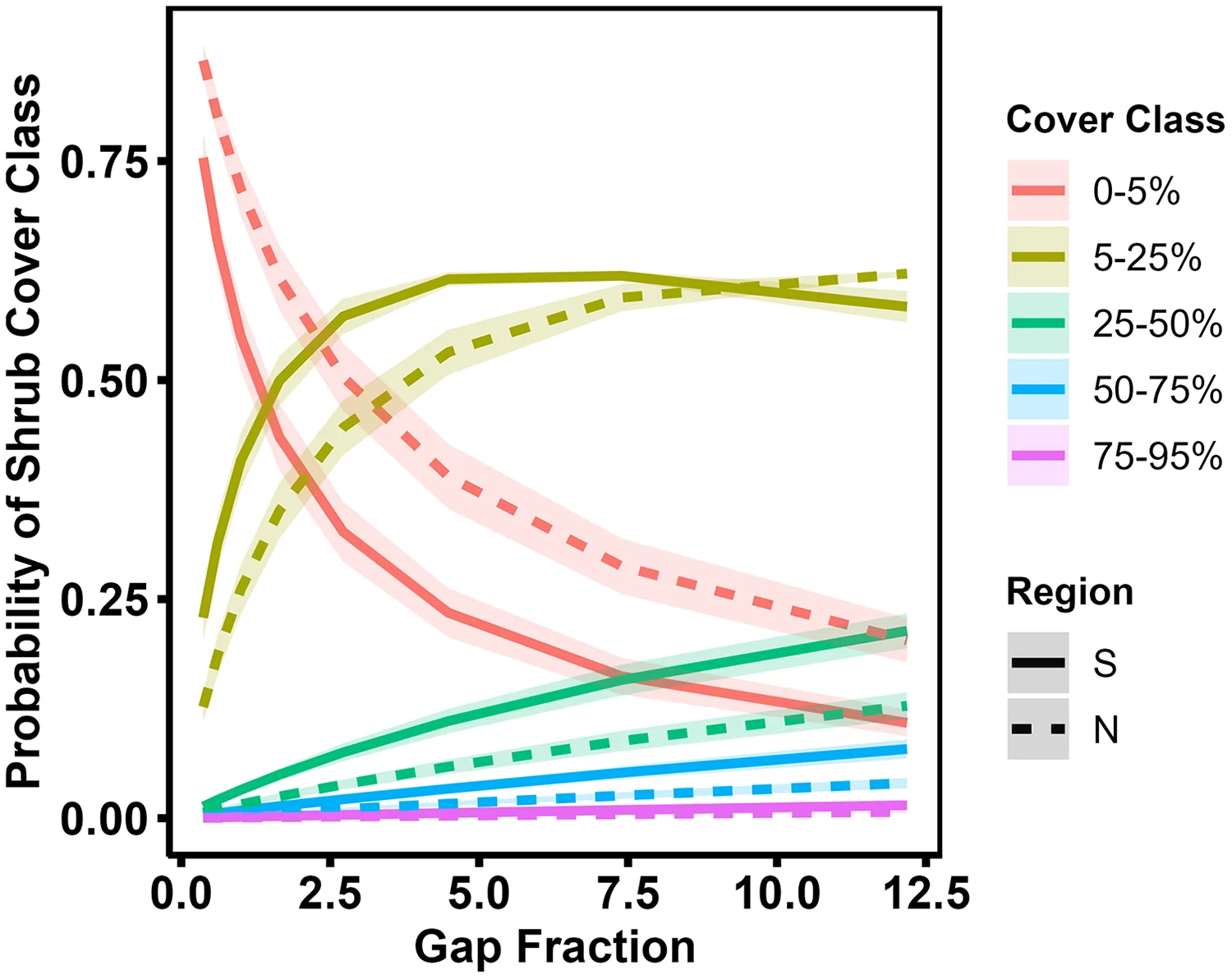
Modelled probabilities of plot cover class along gap fraction gradient. Significant increases in higher cover classes in more open gaps, with the color of each trendline showing different cover classes. Line-types show regional differences in cover class were significant.

Plot level community composition differed between southern and northern communities, though some overlap in species appeared between regions (Fig 3). Environmentally, southern forests experienced higher annual precipitation and mean annual temperature, while northern forests occurred on more sandy substrates and generally had more open canopy gaps (Fig 3 and S1 Table). Within regions, community dissimilarity correlated with A1 horizon depth and gap fraction (Fig 3). PERMANOVA analysis showed that latitude and gap fraction were significantly correlated with NMDS axes, though these environmental variables explained only 1% of the variance in dissimilarity (Fig 3 and Table 1).

**Fig 3:**
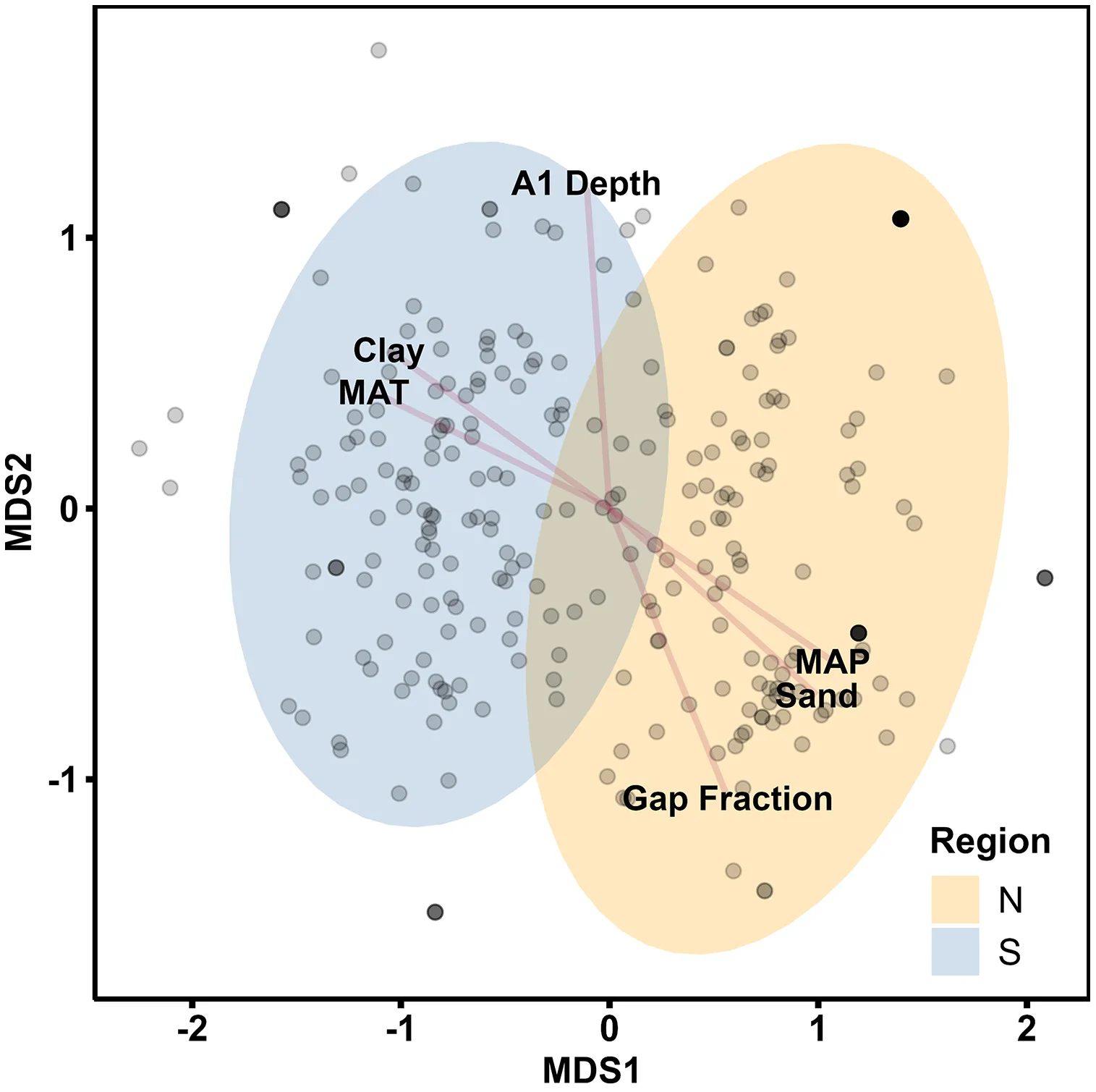
NMDS ordination of plot-level data. An NMDS ordination of plots, with shaded areas denoting northern and southern mesic forests. Environmental vectors correspond to A1 Depth (depth of the A1 soil horizon), Sand (% soil sand content), Gap Fraction (log transformed canopy gap fraction), MAP (20yr mean annual precipitation), MAT (20yr mean annual temperature), and Clay (% soil clay content). Note that darker points reflect occasional overlap in plot composition within sites.

**Table 1:** PERMANOVA results from NMDS ordination. Results of the PERMANOVA analysis of the NMDS ordination in Fig 3, with highlighted rows showing environmental parameters that are significantly correlated with NMDS axes.

	*Df*	*SumOfSqs*	*R* ^*2*^	*F*	*Pr(>F)*	
*Site*	**14**	**36.645**	**0.29923**	**10.6756**	**0.001**	***
*Latitude*	1	0.372	0.00304	1.517	0.09191	.
*Tension*	0	0	0	–	1	
*A1 Soil Depth*	1	0.075	0.00062	0.3074	1	
*Clay*	1	0.181	0.00148	0.7402	0.78621	
*Sand*	1	0.241	0.00197	0.9847	0.43556	
*Gap Fraction*	**1**	**0.407**	**0.00332**	**1.6585**	**0.03197**	*
*Residual*	262	64.238	0.52456			
*Total*	282	122.462	1			

Generally, southern forests sites have a more diverse shrub layer (S2 Fig and S3 Table). However, site level richness and diversity were not correlated with mean site values for gap fraction (S3 and S4 Tables). When considering the variance of gap fractions observed at each site (*i.e.,* canopy heterogeneity), only species richness demonstrated a weak significant relationship (*z =* −2.081, p = 0.03741) with the standard deviation of gap fraction (S4 Table). However, this relationship shows a negative correlation, counter to our hypothesis that more heterogenous forests would support a more diverse shrub layer. Neither mean nor standard deviation values for site-level soil sand content showed any correlation with diversity metrics (S3 and S4 Tables).

### Species hierarchal modelling

The positive posterior distribution for log-transformed gap fraction had a positive impact on shrub presence independent of other plot-level or species effects (Table 2). Posterior distributions for the intercept, sand, and quadratic gap fraction term intersect 0 across models, revealing no general effect on shrub presence, abundance, or coverage in temperate mesic forests. Concurring with our other findings, the explanatory value of microsites assessed was significant, yet minimal (Table 2). At the species level, significant variation was seen in the effects of gap fraction and soil sand content on presence and cover class (Figs 4:6 and S3:S5). For example, *Vaccinium* congeners demonstrated similar, significant preferences for well-lit microsites, while *C. caroliniana* and *V. acerifolium* tended to occupy more shaded understories.

**Table 2:** Results from hierarchal modelling of presence, abundance, and cover class. Fixed and Random Effects from presence/absence models for shrubs. Random effects demonstrate sources of variation within the correlated data structure, and the highlighted rows denote fixed effects whose confidence interval denotes a significant effect on shrub occurrence.

	Occurrence		Abundance (presence only)		Cover Class (presence only)	
*Predictors*	*Odds Ratios*	*CI (95%)*	*Incidence Rate Ratios*	*CI (95%)*	*Odds Ratios*	*CI (95%)*
Intercept	0.02	0.00–0.16	2.42	1.28–4.47		
Gap Fraction	**2.41**	**1.15–5.42**	1.47	0.88–2.49	1.85	0.56–6.29
Gap Fraction^2^	0.87	0.66–1.12	0.93	0.74–1.15	1.04	0.62–1.75
Sand	1.00	0.97–1.03	1.00	1.00–1.01	1.00	0.98–1.02
0-5%|5-25%					**9.90**	**2.55–43.97**
5-25%|25-50					**109.37**	**27.65–512.21**
25-50|50-75%					**397.17**	**92.82–1999.52**
50-75%|75-95%					**2677.73**	**446.03–20840.13**
**Random Effects**						
σ^2^	0.06		32.59		0.19	
τ_00_	0.05		10.88		0.23	
ICC	0.55		0.75		0.46	
N	22 _sp_		22 _sp_		22 _sp_	
	314 _plotid_		16 _site_		16 _site_	
	16 _site_		284 _plotid_		284 _plotid_	
Observations	6864		877		877	
Marginal R^2^/ Conditional R^2^	0.003/ 0.230		0.004/ 0.236		0.013/ 0.262	

**Fig 4:**
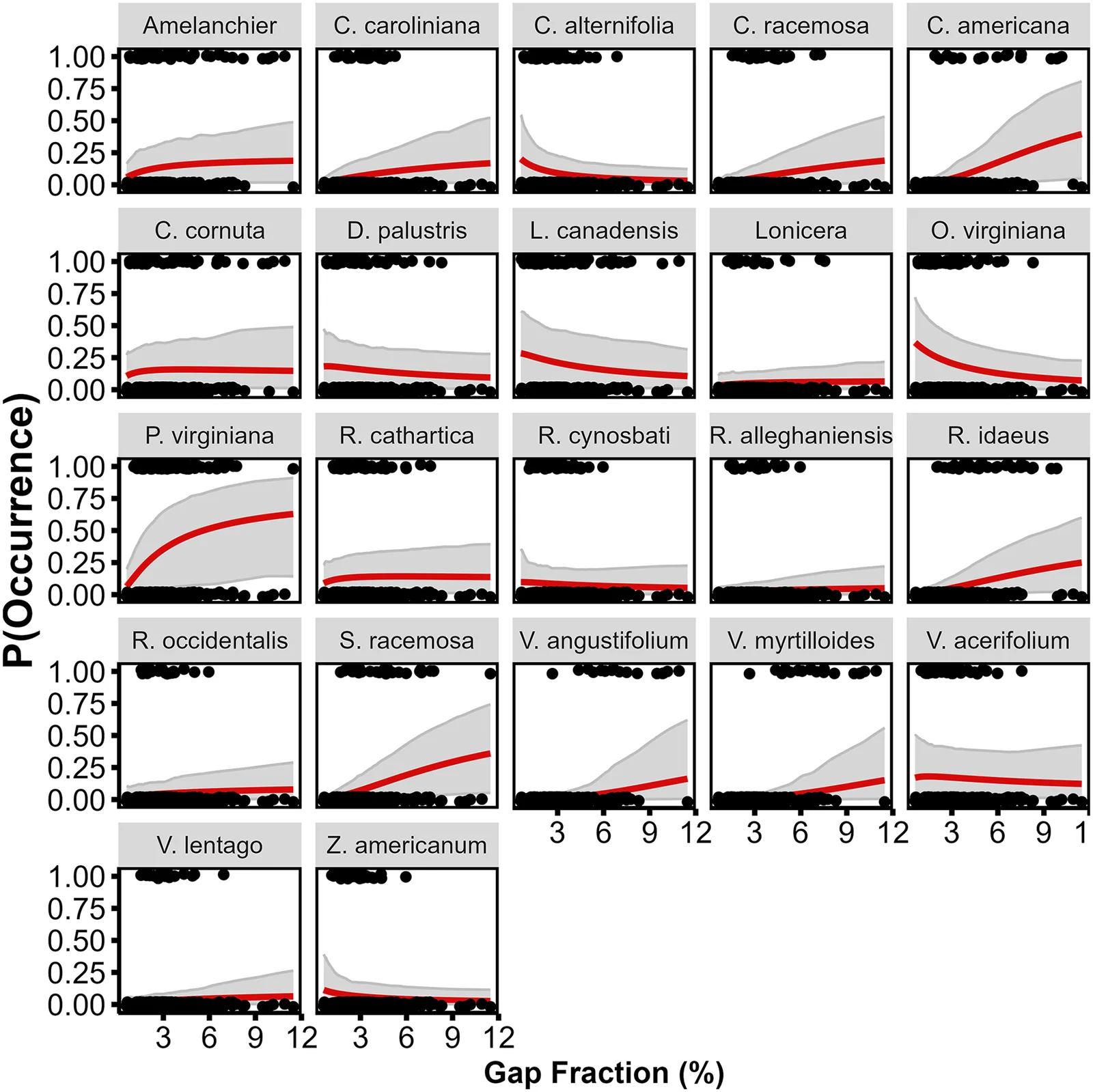
Predicted species occurrence along gap fraction gradient. Predictions of species-level probability of occurrence along gradients of gap fraction. Trendlines and confidence intervals predicted from the model outlined in Table 2, while points show the actual presence/absence values recorded in the field.

Across both plot and species-level models, unquantified site-level effects explained a large portion of model variance. At the plot-level, site random effects explained 54.2%, 58.4%, and 59.2% of the variance in richness, Shannon diversity, and cover class respectively (S2 Table). In species-level hierarchal models, conditional R^2^ also captured 23% of the variance in presence, 23.7% of variance in abundance, and 26.2% variation in cover class (Table 2). Model comparison also highlighted the low explanatory value of plot-level random effects when compared to models excluding important site-level random effects (S6 Table).

## Discussion

### Significant impacts of gaps on shrub communities

Increased shrub community richness, Shannon diversity, and coverage in gaps broadly support our physiologically informed predictions of shrub richness and abundance in temperate understory systems [1,2] (Figs 1 and 2). We also observed significant correlations between community composition and gap fraction (Fig 3). Increased shrub richness in open gaps reflects the impacts of more favorable light environments for this comparatively energetically demanding growth form [2,22]. Since our models involved a log-transformed gap fraction, only minor increases in light availability can have substantial positive impacts on shrub community richness, composition, and abundance in forest understories, corroborated by similar findings in more experimental studies of canopy structure effects on understory richness [75] (Figs 1–6 and Tables 1 and 2). This significance extends to individual shrub species as well, with many species like *Cornus alternifolia* and *Lonicera canadensis* demonstrating an affinity for more close-canopy microsites (Fig 4). These species may possess trait syndromes that allow for persistence in shaded forest understories [14,76,77]. Functional trait and physiological data such as thinner leaves, greater leaf area, and slower growth rates, which are consistent with the acquisition and shade tolerance strategies of individual species could provide further evidence of both competition in brightly lit canopy gaps or niche partitioning by species occupying shadier microhabitats [1,20,78,79]. Our results also provide additional context for shrub layer contributions to understory diversity research, emphasizing the importance of canopy gaps more broadly on all guilds of temperate understory flora [80–82].

**Fig 5:**
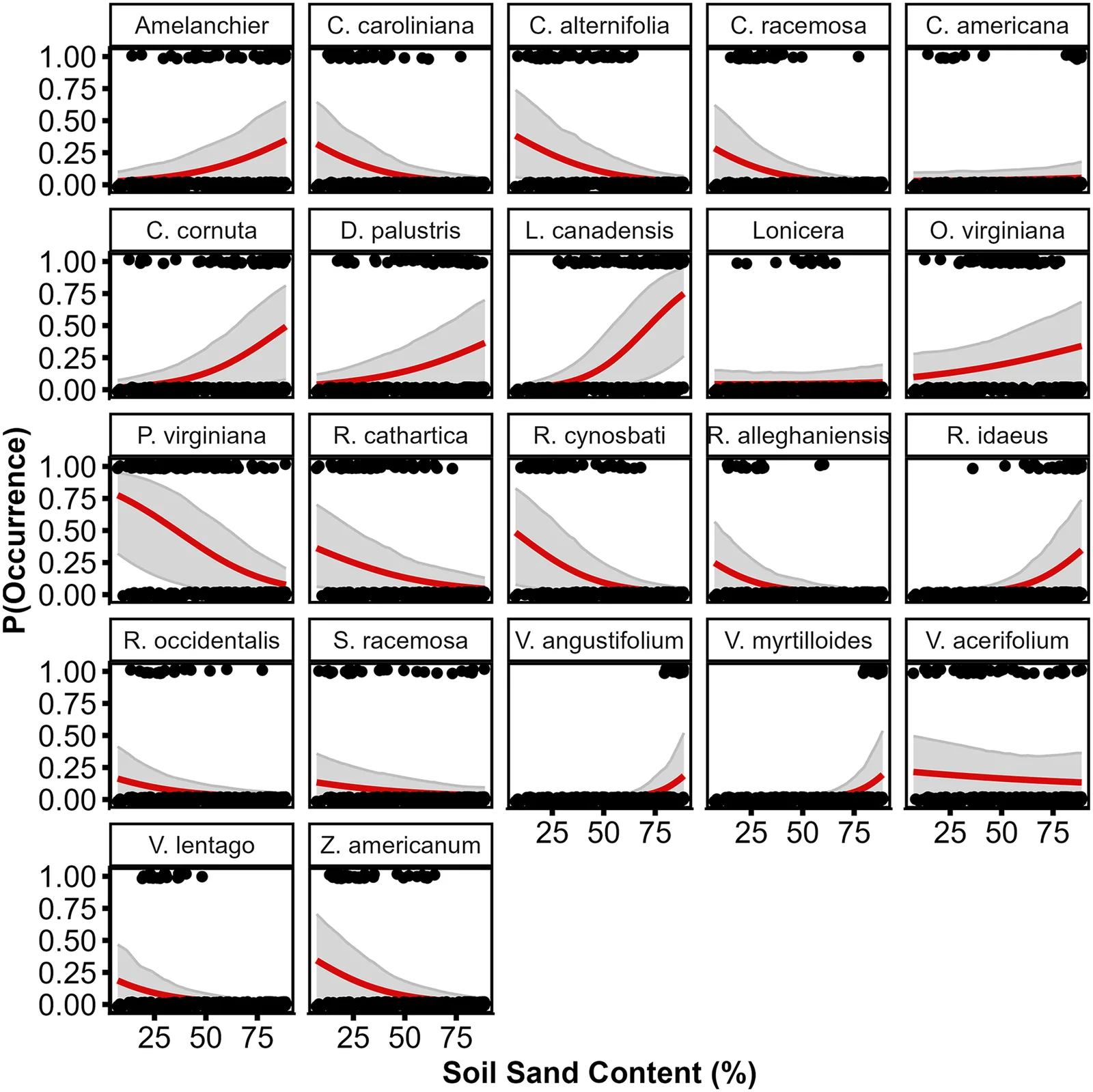
Predicted species occurrence along soil sand content gradient. Predictions of species-level occurrence along gradients of soil sand content. Trendlines and confidence intervals derived from the model outlined in Table 2, while points show the actual presence/absence values recorded in the field.

**Fig 6:**
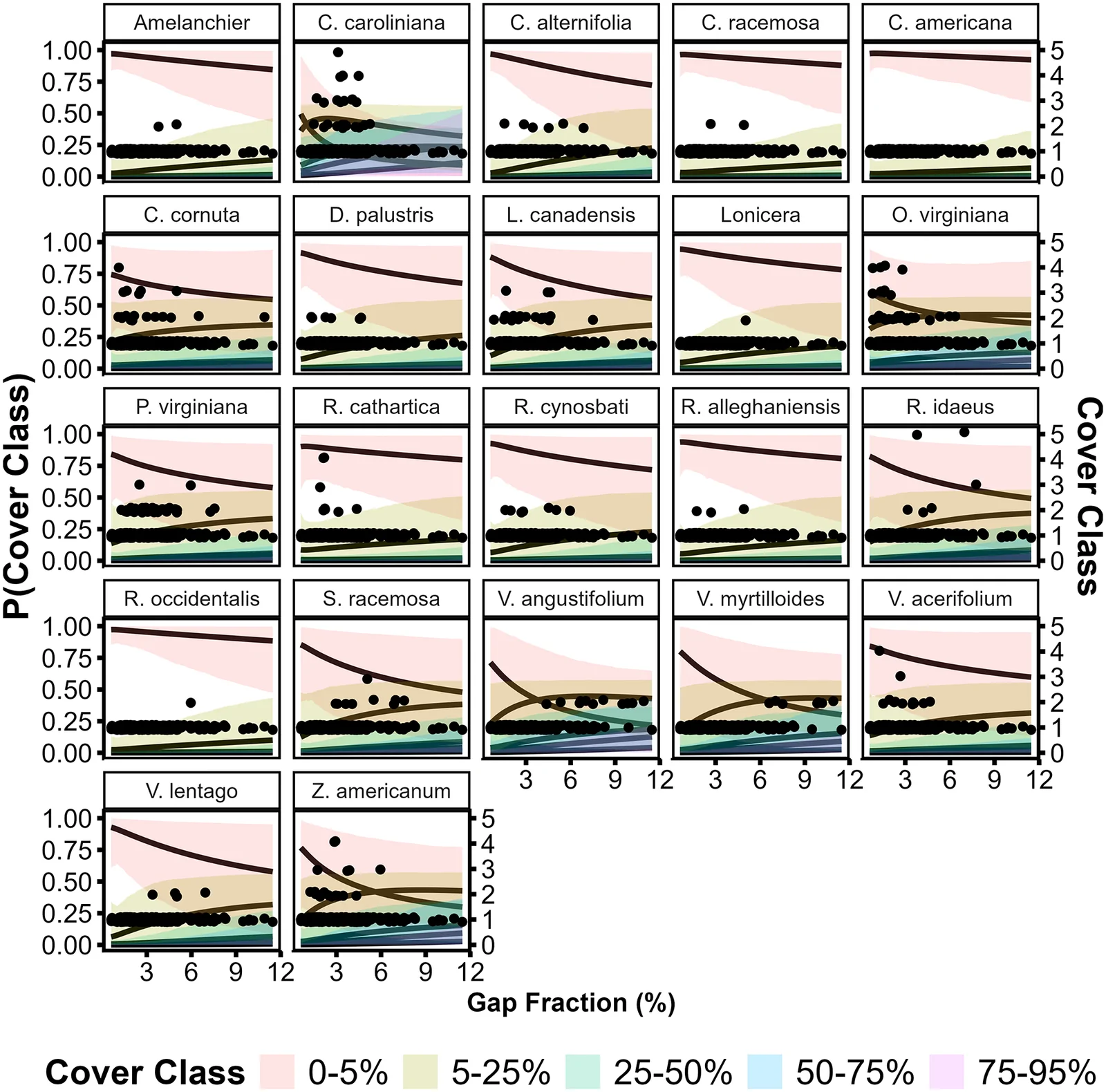
Predicted species cover class along gap fraction gradient. Predictions of species-level probability of a give cover class (assuming presence) along gradients of gap fraction. Trendlines and confidence intervals derived from the model outlined in Table 2, while points show the actual cover class values from survey data.

The less significant relationships between shrub Shannon diversity and gap fraction and differences in predicted shrub cover highlight the patchy dynamics at play in understory shrub communities. When richness is weighted by abundance, more common species in gap microsites reveal disparate preferences for high-light environments (Figs 4, 6 and S3). We see this general propensity towards microsites gaps within our surveying effort with species like *Sambucus racemosa, Rubus idaeus,* and *Corylus cornuta* (Figs 4, 6 and S3). Since some of these species are clonal and readily resprout, growth and competition in response to disturbance are central to understanding species’ affinity for mesic forest gaps, though experimental support for shrubs in particular is sparse [77]. Similar to other studies of soil heterogeneity, we found no significant relationship between plot or site diversity metrics and soil sand content [83] (Tables 2 and S2). This is surprising, considering substantial species-level relationships with soil sand content, (Fig 5 and S5 Table) with species such as both *Vaccinium* congeners*, Lonicera canadensis,* and *Rubus idaeus* demonstrating an affinity for sandier substrates (Fig 5 and S5 Table). Plot-level soil texture varied from 7%−90% (Fig 5), but shrubs may also be accessing resources in deeper than the soil strata analyzed [84], potentially explaining the general lack of correlation between soil texture and shrub communities more broadly. It should also be noted that northern forests had significantly sandier substrates than southern forests (*t* = 5.3298, *df* = 13.935, *p* < 0.001; S1 Table), a confounding factor that potentially impacted species-level predictions related to soil sand content (S3 Fig). Such outputs likely convolute species microsite specificity and species’ distributions along latitudinal gradients. A broader survey of soil textures in both southern and northern forests would improve both model coefficient estimations and address the co-variance of latitude and soil sand content.

### Low explanatory power of gap fraction for shrub communities

Despite the significance of gap microsites on overall richness, coverage, composition, and species-level occurrence, the variance explained by gap fraction was low. The high degree of variability mirrors broader context-dependent interactions between temperate forest canopies and understory communities [85,86], warranting consideration of additional canopy dynamics beyond gap size [87,88](Fig 1 and Table 2). Gap size and light availability are not directly correlated with gap age or successional status, and forest gaps are dynamic, varying drastically in age [14]. Understory vegetation can be impacted by both gap size and gap age, with the effects of gap formation persisting even after canopy closure [14,89]. Herbaceous understory communities in gaps have also been found to respond more to time since disturbance than canopy basal area directly [40]. Older gaps are more likely to carry higher shrub densities and richness than newer gaps in which only the fastest growing species have begun to establish [89–91].

Several analyses specifically indicated that site-level effects improved model fit over plot level effects (Tables 2, S2 and S6), alluding to additional important site-level conditions shaping the structure of understory shrubs beyond the factors we quantified. Broader topographic, and climatic factors have previously demonstrated significant influence on temperate understories [87,88,92]. Microsite heterogeneity and stand structural complexity in the form of nurse logs or pit-mound structures could also impact shrub communities by directly impacting germination and recruitment [93–95]. Specifically within eastern North America, there is also ample evidence that that white-tailed deer (*Odocoileus virginianus*) significantly alter the understory communities of temperate forests [36,90,96–100]. While the impact of browse on native herbaceous species has fostered a contemporary landscape ‘winners’ or ‘losers’ [47] and various tree seedlings have notably been targeted by herbivores [91,101], shrubs that are confined to browsing heights have received less attention. Herbivory is likely also confounded by shrub clonality and resprouting [22,102], with herbivore pressure demonstrating variation with gap size in temperate forest systems [103–105]. Finally, a better understanding of site-level disturbance history may provide additional context for shrub understory dynamics, especially since stand age and management regimes are more often represented in experimental studies focused on understory structure [79,81,82].

Though our analyses demonstrate low explanatory power, our findings align with ongoing limitations in predictive ecology. Community metrics like composition, coverage, and richness are notoriously difficult to predict along ecological gradients like light availability [106,107]. To address this, more complex modelling techniques like hierarchal models of species distributions (HMSC) are being developed to incorporate environmental, functional, and phylogenetic parameters into predictions of community structure and assembly [108–111]. In addition to local light environment, our findings highlight prioritizing the quantification of broader, site-level variables like climate, canopy composition, structural complexity, microtopography, and herbivore pressure [82,112]. These additional climatic, structural, and biotic factors partially contextualize the site-level variance in our analyses while also highlighting the importance of considering external factors at various scales that likely impact the local structure composition of temperate understories.

## Conclusion

The varied light in canopy gap microsites significantly correlates with the presence, abundance, and coverage of shrubs, as well as the overall composition of understory shrub communities in temperate hardwood forests. Soil texture had no significant relation to community composition or shrub diversity metrics, though individual taxa like *Corylus cornuta*, *Amelanchier*, and *Rubus idaeus* did display positive relationships with soil sand content. While we demonstrate significant correlations between canopy structure and shrub communities, shrubs remain highly variable in terms of composition, local abundance, and coverage. Given the overall importance of shrubs to ecosystem services, forest structure, and forest diversity, our results highlight the importance of local light availability in shaping shrub understories. However, the low explanatory value of our analyses alludes to other ecological processes like gap age, gap size, herbivory, and additional site-level factors that are equally important in shaping the shrub community structure of temperate forests.

## Supporting information

S1 Fig Site map. A map of the state of Wisconsin, highlights the location and distribution of the 16 forests used in this study. Base map and data from © OpenStreetMap and OpenStreetMap Foundation. Wisconsin satellite imagery from 2022 provided by Wisconsin Department of Natural Resources through the GeoData@Wisconsin portal.
(TIF)

S2 Fig Species accumulation curves. Species accumulation curves for northern and southern forests sites.
(TIF)

S3 Fig Species abundance predictions along gradient of gap-fraction: Species-level predicted abundances (using left-hand x-axis) along gradient of gap fraction when present shown by red line and grey confidence interval derived from model summarized in Table 2. Dots show recorded abundances at the plot level (using right-hand x-axis).
(TIF)

S4 Fig Species abundance predictions along gradient of soil sand content. Species Species-level predicted abundances (using left-hand x-axis) along gradient of soil sand content when present shown by red line and grey confidence interval derived from model summarized in Table 2. Dots show recorded abundances at the plot level (using right-hand x-axis).
(TIF)

S5 Fig Species cover class predictions along gradient of gap fraction. Species-level predicted cover class (using left-hand x-axis) along gradient of gap fraction when present shown by lines and colored confidence intervals derived from model summarized in Table 2.
(TIF)

S1 Table Site-level environmental metrics. Summary data on location and extracted climatic data from WorldClim, as well as site-level mean soil and canopy metrics calculated from plot-level (n = 20) data collection.
(DOCX)

S2 Table Plot-level mode summaries. Significant terms shown in bold.
(DOCX)

S3 Table Site-level species richness model summary table, with significant terms in bold. (DOCX)

S4 Table Site-level Shannon diversity model summary table, with significant terms in bold. (DOCX)

S5 Table Summary table of environmental conditions for each taxon, with n being the number of individuals observed in surveying. (DOCX)

S6 Table LOO model comparison. Model predictive performance using leave-one-out cross-validation and LOO Information Criterion from the loo function in R package brms (2.23.0). Lower values show better model fit.
(DOCX)

S1 Data Raw data. (XLSX)
