# Supplementary material for: Light availability, not soil texture, correlates with shrub community structure in mesic forest gaps
Source: PLoS One. 2026 Oct 29;21(10):e0361488. doi: 10.1371/journal.pone.0361488 (PMC13718914; doi:10.1371/journal.pone.0361488)
Supplement: Supplementary file 1 [file pone.0361488.s006.docx]

**Table S1: Site-level environmental metrics.** Summary data on location and extracted climatic data from WorldClim, as well as site-level mean soil and canopy metrics calculated from plot-level (n=20) data collection.

| **Site Name** | **Site Code** | **Latitude** | **Longitude** | **MAT (C)** | **MAP (mm)** | **A1 Horizon Depth** | **Clay (%)** | **Silt (%)** | **Sand (%)** | **Gap Fraction (%)** |
| --- | --- | --- | --- | --- | --- | --- | --- | --- | --- | --- |
| Abraham's Woods SNA | AW | 42.683233 | -89.484640 | 7.76 | 1094.53 | 14.76 | 8.19 | 45.81 | 46.00 | 2.28 |
| Baxter's Hollow SNA | BAX | 43.395124 | -89.797804 | 6.97 | 1097.93 | 11.77 | 5.25 | 68.38 | 26.37 | 3.55 |
| Bass Hollow SNA | BH | 43.683840 | -90.027564 | 6.85 | 1106.52 | 11.99 | 4.59 | 54.58 | 40.84 | 2.81 |
| Chequamegon Hardwoods SNA | CH | 46.324409 | -90.788671 | 4.19 | 1136.27 | 13.21 | 2.52 | 42.66 | 54.81 | 1.67 |
| Doering Hardwoods SNA | DH | 45.922576 | -90.106871 | 4.31 | 1142.51 | 9.06 | 2.82 | 18.36 | 78.82 | 4.40 |
| Eagle Heights Woods | EH | 43.084347 | -89.443087 | 7.86 | 1073.77 | 8.34 | 6.81 | 59.81 | 33.38 | 5.04 |
| Kemp Station | KS | 45.839319 | -89.676144 | 4.35 | 1147.87 | 14.85 | 3.53 | 21.48 | 74.98 | 2.82 |
| Lake Laura SNA | LL | 46.050490 | -89.458586 | 3.89 | 1135.25 | 18.58 | 3.33 | 30.41 | 66.27 | 4.40 |
| McGilvra Woods SNA | MW | 43.452271 | -89.815857 | 7.05 | 1101.96 | 13.30 | 8.09 | 72.84 | 19.08 | 1.61 |
| New Observatory Woods SNA | NOW | 43.080325 | -89.672796 | 7.18 | 1098.03 | 12.37 | 7.28 | 69.27 | 23.45 | 3.01 |
| Plum Lake SNA | PL | 46.022172 | -89.464251 | 3.88 | 1135.16 | 6.24 | 3.94 | 10.37 | 85.69 | 7.08 |
| Plum Lake Hardwoods SNA | PLH | 46.194620 | -89.495596 | 3.89 | 1129.33 | 10.03 | 1.51 | 18.71 | 79.77 | 4.50 |
| Raven Lake | RL | 45.870535 | -89.649321 | 4.26 | 1149.38 | 14.09 | 4.13 | 17.80 | 78.06 | 1.72 |
| Saint Peter's Dome SNA | SPD | 46.348834 | -90.918778 | 4.43 | 1136.48 | 16.25 | 2.72 | 50.58 | 46.70 | 2.15 |
| Texas Island Woods SNA | TI | 42.981180 | -88.585615 | 7.94 | 1073.30 | 18.66 | 4.33 | 38.28 | 57.39 | 2.34 |
| Wingra Woods | WW | 43.046188 | -89.429189 | 7.95 | 1098.20 | 14.30 | 7.06 | 54.69 | 38.25 | 2.61 |
