# Supplementary material for: Light availability, not soil texture, correlates with shrub community structure in mesic forest gaps
Source: PLoS One. 2026 Oct 29;21(10):e0361488. doi: 10.1371/journal.pone.0361488 (PMC13718914; doi:10.1371/journal.pone.0361488)
Supplement: Supplementary file 2 [file pone.0361488.s007.docx]

**Table S2:** Plot-level mode summaries. Significant terms shown in bold.

|  | **Richness** | | **Shannon Diversity** | | **Daubenmire Cover Class** | |
| --- | --- | --- | --- | --- | --- | --- |
| *Predictors* | *Incidence Rate Ratios* | *CI* | *Estimates* | *CI* | *Odds Ratios* | *CI* |
| Intercept | **2.31 ^*^** | **1.22 – 4.36** | **0.67 ^**^** | **0.22 – 1.12** |  |  |
| Gap Fraction | **1.23 ^*^** | **1.04 – 1.47** | **0.11 ^*^** | **0.00 – 0.22** | **2.51 ^***^** | **2.50 – 2.52** |
| Sand Content | 1.00 | 0.99 – 1.00 | -0.00 | -0.01 – 0.00 | 1.00 | 1.00 – 1.00 |
| Region [S] | 1.18 | 0.65 – 2.14 | 0.16 | -0.29 – 0.61 | **2.09 ^***^** | **2.08 – 2.09** |
| 0-5%\|5-25% |  |  |  |  | **2.78 ^***^** | **2.77 – 2.79** |
| 5-25%\|25-50 |  |  |  |  | **51.28 ^***^** | **51.12 – 51.43** |
| 25-50\|50-75% |  |  |  |  | **220.26 ^***^** | **219.58 – 220.94** |
| 50-75%\|75-95% |  |  |  |  | **1520.53 ^***^** | **1515.87 – 1525.21** |
| **Random Effects** | | | | | | |
| σ^2^ | 0.27 | | 0.14 | | 3.29 | |
| τ_00_ | 0.29 _site_ | | 0.18 _site_ | | 4.44 _site_ | |
| ICC | 0.52 | | 0.56 | | 0.57 | |
| N | 16 _site_ | | 16 _site_ | | 16 _site_ | |
| Observations | 314 | | 314 | | 314 | |
| Marginal R^2^ / Conditional R^2^ | 0.046 / 0.542 | | 0.055 / 0.584 | | 0.043 / 0.592 | |
| ** p<0.05   ** p<0.01   *** p<0.001* | | | | | | |
