# Supplementary material for: Light availability, not soil texture, correlates with shrub community structure in mesic forest gaps
Source: PLoS One. 2026 Oct 29;21(10):e0361488. doi: 10.1371/journal.pone.0361488 (PMC13718914; doi:10.1371/journal.pone.0361488)
Supplement: Supplementary file 3 [file pone.0361488.s008.docx]

**Table S3:** Site-level species richness model summary table, with significant terms in bold.

|  | **SR** | | **SR** | | **SR** | | **SR** | |
| --- | --- | --- | --- | --- | --- | --- | --- | --- |
| *Predictors* | *Incidence Rate Ratios* | *CI* | *Incidence Rate Ratios* | *CI* | *Incidence Rate Ratios* | *CI* | *Incidence Rate Ratios* | *CI* |
| Intercept | **10.86 ^***^** | **4.19 – 27.30** | **7.65 ^***^** | **5.37 – 10.76** | **4.61 ^***^** | **1.90 – 10.93** | **13.76 ^***^** | **7.24 – 25.40** |
| Sand Content | 0.99 | 0.98 – 1.01 |  |  |  |  |  |  |
| St.Dev (Sand Content) |  |  | 0.99 | 0.96 – 1.03 |  |  |  |  |
| Gap Fraction |  |  |  |  | 1.29 | 0.82 – 1.99 |  |  |
| Std.Dev (Gap Fraction) |  |  |  |  |  |  | **0.16 ^*^** | **0.03 – 0.90** |
| Region [S] | 1.31 | 0.76 – 2.30 | **1.63 ^**^** | **1.14 – 2.35** | **1.66 ^**^** | **1.19 – 2.34** | **1.69 ^**^** | **1.22 – 2.37** |
| Observations | 16 | | 16 | | 16 | | 16 | |
| R^2^ Nagelkerke | 0.482 | | 0.457 | | 0.504 | | 0.616 | |
| ** p<0.05   ** p<0.01   *** p<0.001* | | | | | | | | |
