# Supplementary material for: Light availability, not soil texture, correlates with shrub community structure in mesic forest gaps
Source: PLoS One. 2026 Oct 29;21(10):e0361488. doi: 10.1371/journal.pone.0361488 (PMC13718914; doi:10.1371/journal.pone.0361488)
Supplement: Supplementary file 4 [file pone.0361488.s009.docx]

**Table S4:** Site-level Shannon diversity model summary table, with significant terms in bold.

|  | **H** | | **H** | | **H** | | **H** | |
| --- | --- | --- | --- | --- | --- | --- | --- | --- |
| *Predictors* | *Estimates* | *CI* | *Estimates* | *CI* | *Estimates* | *CI* | *Estimates* | *CI* |
| Intercept | 1.83 | -0.01 – 3.67 | **1.36 ^***^** | **0.69 – 2.03** | 0.76 | -0.84 – 2.36 | **1.79 ^**^** | **0.54 – 3.04** |
| Sand Content | -0.01 | -0.03 – 0.02 |  |  |  |  |  |  |
| St.Dev (Sand Content) |  |  | -0.02 | -0.10 – 0.06 |  |  |  |  |
| Gap Fraction |  |  |  |  | 0.27 | -0.57 – 1.10 |  |  |
| Std.Dev (Gap Fraction) |  |  |  |  |  |  | -1.57 | -4.97 – 1.83 |
| Region [S] | 0.09 | -0.99 – 1.18 | 0.46 | -0.26 – 1.17 | 0.42 | -0.21 – 1.06 | 0.43 | -0.19 – 1.05 |
| Observations | 16 | | 16 | | 16 | | 16 | |
| R^2^ Nagelkerke | 0.147 / 0.015 | | 0.130 / -0.004 | | 0.145 / 0.014 | | 0.178 / 0.051 | |
| ** p<0.05   ** p<0.01   *** p<0.001* | | | | | | | | |
