# Supplementary material for: Light availability, not soil texture, correlates with shrub community structure in mesic forest gaps
Source: PLoS One. 2026 Oct 29;21(10):e0361488. doi: 10.1371/journal.pone.0361488 (PMC13718914; doi:10.1371/journal.pone.0361488)
Supplement: Supplementary file 5 [file pone.0361488.s010.docx]

**Table S5:** Summary table of environmental conditions for each taxa, with n being the number of individuals observed in surveying.

| **Taxa** | **Mean Gap Fraction** | **St. Dev Gap Fraction** | **Mean Sand Content** | **St. Dev Sand Content** | **n** |
| --- | --- | --- | --- | --- | --- |
| *Amelanchier* | 4.26 | 2.88 | 65.02 | 20.95 | 45 |
| *Carpinus caroliniana* | 3.316 | 0.99 | 31.87 | 14.81 | 32 |
| *Cornus alternifolia* | 2.52 | 1.29 | 34.09 | 15.74 | 45 |
| *Cornus racemosa* | 3.61 | 1.43 | 30.43 | 12.82 | 27 |
| *Corylus americana* | 5.84 | 2.74 | 59.87 | 30.75 | 20 |
| *Corylus cornuta* | 3.81 | 2.53 | 66.68 | 19.49 | 55 |
| *Dirca palustris* | 3.05 | 1.71 | 60.21 | 20.24 | 50 |
| *Lonicera canadensis* | 3.42 | 2.11 | 69.60 | 16.21 | 88 |
| *Lonicera* | 3.39 | 2.061 | 49.44 | 13.66 | 15 |
| *Ostrya virginiana* | 2.66 | 1.47 | 53.99 | 13.67 | 65 |
| *Prunus virginiana* | 3.38 | 1.68 | 38.12 | 17.66 | 112 |
| *Rhamnus cathartica* | 2.98 | 1.46 | 36.42 | 15.98 | 50 |
| *Ribes cynosbati* | 2.73 | 1.14 | 31.05 | 16.48 | 46 |
| *Rubus alleghaniensis* | 3.17 | 1.24 | 27.30 | 13.31 | 18 |
| *Rubus idaeus* | 5.40 | 2.14 | 77.54 | 12.06 | 34 |
| *Rubus occidentalis* | 3.09 | 1.15 | 34.38 | 15.63 | 19 |
| *Sambucus racemosa* | 4.50 | 2.36 | 41.92 | 26.41 | 26 |
| *Vaccinium angustifolium* | 7.19 | 2.22 | 85.87 | 2.812 | 18 |
| *Vaccinium myrtlloides* | 7.11 | 2.23 | 85.63 | 2.912 | 19 |
| *Viburnum acerifolium* | 2.81 | 1.33 | 45.00 | 21.12 | 53 |
| *Viburnum lentago* | 3.31 | 1.42 | 29.74 | 8.15 | 16 |
| *Zanthoxylum americanum* | 2.54 | 1.07 | 32.047 | 16.10 | 34 |
