# Supplementary material for: Light availability, not soil texture, correlates with shrub community structure in mesic forest gaps
Source: PLoS One. 2026 Oct 29;21(10):e0361488. doi: 10.1371/journal.pone.0361488 (PMC13718914; doi:10.1371/journal.pone.0361488)
Supplement: Supplementary file 6 [file pone.0361488.s011.docx]

**Table S6:** Model predictive performance using leave-one-out cross-validation and LOO Information Criterion from the *loo* function in R package *brms* (2.23.0). Lower values show better model fit

| **Model** | **Abundance** | **Occurrence** | **Coverage** |
| --- | --- | --- | --- |
| Full | 4458.5 | 3934.0 | 1168.7 |
| No Site Random Effect | 4531.8 | 4149.5 | 1210.6 |
| No Plot Random Effect | 4454.2 | 3932.1 | 1166.7 |
