# Supplementary material for: Light availability, not soil texture, correlates with shrub community structure in mesic forest gaps
Source: PLoS One. 2026 Oct 29;21(10):e0361488. doi: 10.1371/journal.pone.0361488 (PMC13718914; doi:10.1371/journal.pone.0361488)
Supplement: Supplementary file 7 [file pone.0361488.s013.docx]

To the Editor and our Reviewers,

Thank you for the opportunity to revise and resubmit our manuscript, retitled “Light availability, not soil texture, correlates with shrub community structure in mesic forest gaps” to PLOS One. Your feedback and comments were incredibly helpful in honing the framing of this observational study while also contextualizing our findings. We hope our introduction provides a more concrete rationale for pursuing this study and our results more accurately reflect the correlative, descriptive outcomes of our analysis. Both reviewers highlighted our initial omission of shrub coverage data, so we hope our detailed response and updated analyses assuage your concerns. We also reshaped our figures in the manuscript and supplemental to better communicate our main conclusions.

With your feedback, we believe our manuscript better reflects our initial motivations, accurately portrays our main findings, and stands as a solid contribution to both PLOS One and our understanding of temperate understory shrub communities.

Thank you again and all the best,

Sam Anderson

**Response to Comments**

Below are our responses to both general comments and line-item revisions. Our responses are italicized and include updated line numbers for your reference were applicable.

We want to address the omission of coverage data from our original manuscript. Cover data (% cover) for each observation was recorded in the field, but modelling % cover directly resulted in issues with model convergence/performance. Instead, we focused our attention initially on occurrence and abundance for our submitted manuscript. In response to your feedback, we pursued the analysis of ordinal cover class for both shrubs in general and individual species.

In addition to feedback from you and other colleagues, we have also adjusted our modelling approach. We now model abundance and coverage on presence-only data to separate out the impacts of environmental variables on presence vs. cover when present, and we now include a quadratic term and omit observation-level random effects to address potential modal distributions observed in the raw data and to improve model performance, respectively. These efforts have not substantively altered our main takeaways, but we believe they make for cleaner, more concise model summaries that better reflects our observational data.

We have made major updates to our figures and tables to address your feedback and better communicate our findings. This includes new figures to show cover class models, supplemental tables to better summarize model outputs, and more interpretable species-level plots that bolster descriptive patterns in the text. Thank you for your insight in making our work more interpretable!

**Response to Editor’s general feedback**

strengthening the framing of the study

*In our introduction, we included more context for our study system (updated lines 50-55) and more directly outlined our rationale for hypotheses (updated lines 114-121) related to soil sand content (updated lines 86-91). With more concise language, we hope the context for our study is stronger and more direct.*

clarifying the methodological details,

*We hope the inclusion of site selection criteria (updated lines 124-129, 131-141), descriptions of model (updated lines 171-173,187-193) distributions, and other edits to the methods (updated lines 155-159) meet your expectations.*

acknowledging the limitations more explicitly

*Thank you for this. We have updated the abstract (updated lines 43-44), results (updated lines 258-265), discussion (updated lines 316-331, 348-351), and conclusion (updated lines 365-368) to better reflect our observational study and the caveats that go along with our chosen study design.*

adopting a more cautious and descriptive interpretation of the results

*We appreciate the reminder to avoid causal language in observational studies such as ours. We have updated phrasing in our title, abstract results, discussion, and conclusions (see above subsection line edits) to be more correlative and descriptive, and juxtaposed our findings by describing the importance of experimental studies that would strengthen our* in-situ *findings*

**Reviewer #1: Comments**

I believe the research idea itself has considerable potential, and the study provides, in general, suitable data to address the two main hypotheses. However, the applied field methodology substantially constrains the extent to which these questions can be meaningfully explored. Although the sampling sites are distributed across a relatively large area and include a north–south comparison, the overall sample size and survey effort appear limited relative to the likely high environmental heterogeneity. Key contextual information is lacking, such as clear characterization of forest types, more detailed description of canopy gaps beyond light availability and a more comprehensive approach to soil sampling. As a result, it is difficult to account for substantial environmental “noise” across sites.
Given these limitations, I am skeptical that causal conclusions can be drawn from the current dataset. While it may still be informative to report that shrub species richness tends to be higher in sandier and more open (light-rich) environments compared to more closed and loamy conditions, the current evidence does not appear sufficient to support stronger interpretations.

A further concern is the absence of vegetation cover data, which is a standard and important component of understory vegetation studies, particularly when sampling intensity is relatively low. While presence–absence data have a long tradition in vegetation ecology, and stem counts can provide additional information, these metrics alone may not be sufficient for the stated research aims.

*Thank you for this comment. We hope the inclusion of cover class analysis (updated lines 135-140, 172-173, 181-193 for methods) (Fig. 2,4,5, Tables 2,S2) addresses your concerns.*

On the positive side, the statistical approaches are well executed and sophisticated, and it would be highly interesting to see how such models perform on a dataset with more detailed vegetation structure and environmental data.

*Thank you for this, we hope our new figures better present the outputs of our modelling approach, especially in relation to individual species and our cover class data (Figs 3,4,5).*

Abstract

The paragraph is generally well written; however, it places too much emphasis on general background rather than the research itself. Approximately half of the abstract reads like an introduction, whereas it should focus more directly on the study. A brief contextual lead-in of one or two sentences would be sufficient, followed by a clearer statement of the research purpose. Greater emphasis should then be placed on the methods, key findings, and their implications.

*We shortened the contextual portion of the introduction to 1-2 sentences (updated lines 26-22), and better described our methods (updated lines 33-34), hypotheses (updated lines 32-33), and major takeaways (updated lines 34-41).*

Introduction
Another major disturbance factor that could be considered is the impact of climate change on ecosystems worldwide. This driver increasingly overrides many natural dynamics, including gap formation and successional patterns. However, this is intended as a suggestion rather than a critical omission.

*We agree with your perspective on climate change. In fact, we are currently using	historical datasets on shrub/sapling structure in Wisconsin to tease apart the effects	of successional patterns and climate change. We hope to have this work in review	by the end of the summer!*

While the importance of light availability is well addressed in the text, it may be helpful to briefly explain why soil sand content is also a determining factor. This would make lines 107–108 easier to understand. In general, soil sand content refers to the proportion of particles approximately 0.05–2 mm in size, depending on the classification system used. In many ecosystems with highly sandy soils, however, its role can be more complex or even controversial, as rapid water percolation limits the time available for plant uptake. In such cases, higher sand content may have negative effects on vegetation diversity, including shrubs.

*Thank you for this feedback. We have added more context for the role of soil sand	content in shaping shrub communities. In particular, we reference this in our Abstract (31), the introduction (50-52, 85-91), and our more clearly written hypotheses (116-121).*

The use of more literary expressions such as “myriads” or “a plethora of” might be reconsidered. Although they can improve readability, they may be less appropriate in a scientific context, where more neutral and precise wording is generally preferred.
The aims of the study, including the hypotheses, should be highlighted in a separate, clearly structured list. Otherwise, the chapter is well written, and the topic and the subsequent research are introduced sufficiently well.

*We hope our structured hypotheses (116-121) and more direct, scientific phrasing make	for a more concise and direct Introduction.*

Methods

It is not stated in the main text that 16 forest sites were sampled (this information appears only in Supplementary Fig. 1). This should be clearly highlighted, along with the total number of samples collected. As it stands, the reader must infer these values: assuming 16 forest sites with 20 samples each, this would amount to 320 samples in total. Given that each sample covers 50 m², the total sampled area would be 16,000 m2 (1.6 ha). If this interpretation is correct, then despite the sites being distributed across several forest stands, the total sampled area appears relatively small in relation to the study’s aims.

*We hope the inclusion of this information in the abstract (33-34) and methods (131-135). While the sum total area surveyed is small, our research questions were directly focused on characterizing the impacts of microsites within temperate forest understories that are inherently shaped by canopy structure. Future work aims to expand this work to more open forest and savanna sites in the region*

Another issue is that very little information is provided about the canopy gaps themselves. It is unclear whether these are typical small-scale gaps (e.g., on the order of one to two tree lengths) or whether they represent quantified canopy gap fractions. As a result, it is difficult for the reader to form a picture of what these gaps actually looked like in the field. While the data handling of gap metrics is described in detail, a more explicit characterization of the gaps (e.g., their size, structure, and typical appearance) would greatly help the readers.

*Thank you for this reminder. We hope our addition of more description of canopy gaps (57-58,156-159) compliments our methods describing gap metrics.*

Another aspect that is lacking is a clear introduction to the study site. Key information such as soil characteristics, mean annual temperature and precipitation, potential north–south gradients, and other environmental factors is not provided. In addition, details on forest types and land-use history would be valuable. Without this context, readers – particularly those unfamiliar with the region – are left with only a vague understanding that the study was conducted in a temperate forest.

*We hope our expanded descriptions of site conditions and variation (50-58, 127-129, Table S1) provide a more detailed picture of temperate mesic forests.*

A final issue with the field methods concerns the sampling depth, as soil was collected only from the top 15 cm. While these shrub species can likely reach this depth within one or a few years, the success of individual plants is largely determined by root competition, which typically extends well beyond this layer. Consequently, soil texture was characterized only for the uppermost horizon, raising questions about its ecological relevance. In particular, plant performance may depend strongly on deeper soil layers and the properties of the (full) A and other (particularly B) horizons, which were not considered here.

*Thank you for this. We agree that our soil sampling of the A1 horizon doesn’t capture the entirety of the substrate environment. The constraints of texture analysis for over 300 plots (tests take ~7hrs) limited our ability to sample from multiple soil strata per plot. Species-level trends with soil sand content (Fig 5, Table S5) demonstrate that A1 soil texture is still ecologically relevant, but our modelling outputs do not support more general effects on shrub communities. We also address this limitation in our revised discussion (308-310)*

L124–125: Regarding the choice of survey locations, it would be useful to clarify whether site selection was conducted in a GIS environment (i.e., in a semi-blinded manner) or based on prior visual assessment and subsequent random selection in the field. Both approaches are valid, but this part should be specified.

*This was a straightforward revision that was easily addressed (129-133)*

L127–131: The decision not to record vegetation cover is questionable. Cover is a particularly important metric for the investigated species, especially for clonal taxa (e.g., Rubus, Ribes) or species where multiple stems originate from a single rootstock (e.g., Crataegus). More generally, these species can occupy several square meters even as a single individual. Although abundance is counted as one when stems originate from the same individual, the lack of cover data may introduce substantial noise into the results, particularly when assessing species richness or diversity in a heterogeneous understory. Including cover estimates would likely provide more informative and ecologically meaningful data than presence and abundance.

*Thank you for this feedback, and we agree completely. We hope our inclusion of cover class data directly address your concerns.*

The statistical analyses are appropriate and perhaps even somewhat overly detailed. However, the inclusion of code in the main text is unnecessary and should be reconsidered. A more important question is whether the collected data (small sample size, and abundance without coverage) are suitable for this type of analysis. Nonetheless, I appreciate the range of statistical methods used by the authors.

*Thank you for this! Our updated modelling is more streamlined than the previous manuscript (189-193), and we adopted more verbal descriptions of models as opposed to including code directly in the text.*

Results

Fig. 1 – The image appears to be poorly edited in the version I downloaded, although this may be due to the downloading process and not reflect the original quality. Currently, all points are displayed in the same color (green), with a yellow boundary line. Important cartographic elements such as scale, resolution, and a north arrow are missing. In addition, several points overlap, reducing clarity. This could be improved by separating the northern and southern parts into distinct panels and then combining them into a single composite figure. Including proper scaling and additional reference features (e.g., forest stand boundaries or nearby settlements for orientation) would further enhance interpretability

*We hope our updated make is easier to interpret. Though separately mapping regions would provide more local context for study sites, we believe that a single map serves to orient readers, especially those who appreciate larger, regional context.*

Overall, the results section is concise and informative, and the figures are adequate and clearly present the findings. However, the frequent occurrence of non-significant results, high standard deviations, and low explained variance raises questions about whether these patterns may be attributable to the study design itself, particularly the limited sample size and the absence of other vegetation measures. The 16 sites are also likely relatively far apart and situated in presumably different forest types, which may introduce substantial environmental heterogeneity and additional noise that is difficult to account for.

*Thank you for this feedback. We previously acknowledged the common result of	observational studies failing to account for much of the variation in local plant	occurrence/cover. In particularly, our site-level analyses were broadly insignificant,	which we believe to be better summarized in our revised supplemental (Tables S3,S4).	With our main	motivation being to identify shrub community and species-level	correlations with local micro-site conditions, our findings do provide both community	and species-level significant relationships (Fig 1,2,3 Tables 1,2). Our statistical analyses	also highlight the importance of site-to-site variation, which we hope we now address	more directly in our results (258-264) and discussion (318-348).*

Discussion

The first subchapter relates the study’s findings well to those of similar research. In contrast, the second subchapter contains rather general statements, particularly in lines 302–333, that do not appear to be well supported by the present results. While I agree that the mentioned factors can influence shrub communities alongside several other drivers, this discussion would be more appropriately placed and briefly addressed in the introduction. The inclusion of such broader statements here may reflect the lack of strong results, and I am concerned that this is primarily attributable to the limited sampling effort.

*We understand your feedback, and we hope re-framing our introduction and discussion	address the generality of our statements and the inclusion of unquantified drivers. Our	modelling results highlight that local light environment can have significant impacts of	community structure and species presence (Figs 1,2,4; Table 2), but these local	environmental effects are nested within a large degree of site-level variation (Table	1,2,S2,S6). We felt that this warranted a discussion of additional site-level drivers that	contextualize our findings, which did demonstrate statistically significant correlations	that are informative, especially at the species level (Figs 4,5,6).*

Conclusion
It is again rather general and should focus more specifically on how this research contributes to our understanding of shrub communities in deciduous forest ecosystems within temperate climates.

*Thank you for this, we hope our more targeted language makes for a more concise	summation of the contributions of this observational study.*

**Reviewer #2: General evaluation of the MS**
The study is well-framed around canopy gaps, light availability and soil texture, assumed to be key drivers in mesic forest ecology. It clearly articulates why understory shrubs matter, which helps justify the study. Multi-scale perspective (plot vs. stand level) is also a relatively strong point, although it should be more specified in the Abstract.

*Thank you for this, our updated abstract now references our site-level analysis (revised lines 33-34).*

This study brings some insight into the dynamics and ecological determinants of shrub layer communities in Wisconsin’s mesic forests, where shrubs represent ~60% of the woody flora and shade is presumably the major driver of diversity and community composition. Authors analyzed shrub layer communities with respect to light availability (gap fraction) and soil conditions (sand content in top mineral horizon). They found that light (presence of canopy gaps) is more important, while soil sand appeared to be more species-specific driver. Authors discuss the low explanatory power of used predictors and acknowledge that other abiotic (e.g. heterogeneity in light and soil conditions) or biotic (deer herbivory) factors not accounted for are likely even more important in shaping shrub communities under mesic canopies in hardwood forests. I was a bit disappointed to see that only light and soil sand were included. I wonder why other variables were not inventoried at sampling plots? Some of them could be obtained with quick visual examination, no? It feels like a missed opportunity.

*We agree that additional visual estimates of plot-level variables (i.e. tip-up mounds,	browsing damage etc.) would have been interesting to include. We hope to address these	metrics in future surveys of a broader range of forest community types in the future.*

In my opinion, one of the main weaknesses are the Title and the Abstract. For the Title: It is clear and accurate, but slightly broad and generic. “Microsite specificity” is somewhat vague and could be more explicit. My suggestion would be to make it more specific and outcome-oriented, e.g. something like “Canopy gaps drive shrub diversity and species-specific microsite preferences in mesic forests” or “Light availability, not soil texture, shapes shrub community structure in mesic forest gaps”

*Thank you for this feedback. We agree that our title is more descriptive than specific, but we also wanted to be wary of explicitly drawing causal conclusions from the results of our observational study. We hope the updated title is satisfactory for all reviewers.*

For the Abstract:

(1) The background is slightly wordy and could be tightened.

*Thank you for this. We have tightened up the background section to 1-2 sentences (26-30), and expanded upon our methods, hypotheses, and main findings (32-41).*

(2) More importantly, the abstract lacks even minimal detail on how the study was conducted (e.g., sampling design, number of plots, metrics). And

*This was solid feedback that we readily incorporated (33-34).*

(3) The conclusion leans on broad ecological ideas without clearly stating what’s novel. For instance, “Our results support previous hypotheses…” is too broad so I recommend trying something more assertive e.g. “These findings highlight light availability as the primary driver of shrub community assembly in mesic forests, while species-specific responses to microsite conditions reveal fine-scale niche partitioning that is not captured at broader spatial scales.”

*Thank you for this feedback. We hope our new phrasing avoids generalities and directly ties our novel findings to our broader understanding of forest understories, but we also want to be aware of being overly-causal with our findings. Our results demonstrate that canopy gap fraction is significantly correlated with many metrics of shrub communities, but the large amount of site-level variation and in our models makes us wary of labelling light as a primary driver. Your species-specific comment is well places and readily included (361-362).*

Regarding the methodological approach, soil sand content alone is a very limited proxy for soil conditions. Authors should clearly state this limitation in the Discussion section. Shrubs are likely impacted by other soil variables, such as soil moisture, organic matter, nutrient availability (N, P) and/or at least soil pH (usually strong effect on plant occurrence patterns).

*Thank you for this, we hope our updated methods reflected the limitation of sand content	as a proxy measure of soil conditions (85-89). With our focus on local plot effects	(n=320), nutrient analysis was cost-prohibitive, though soil pH can easily be included in	future studies.*

Specific comments
L36: What type of diversity, which metric? Please be more specific.

*Shannon diversity is more explicitly labelled throughout the manuscript.*

L71: Woody growth also means deeper rooting (compared to herbaceous species), making it less susceptible to summer drought (if they occur in mesic forests).

*We included deeper rooting and comparisons to herbaceous plants specifically to	better characterize shrub allometry (71-73,308-310)*

L75-79: That is solid justification of why shrubs are important to study, yet often overlooked. More focus has been placed on trees (e.g. economic value of a forest) and herbs (main diversity assets in temperate forests).

*Thank you! We are glad to hear we provided good context for our study!*

L87: Here the authors address soil moisture availability and soil fertility (productivity), yet these were not included in their approach. They solely rely on soil texture. I think a stronger explanation why this approach seems reasonable is needed in the Introduction.

*Thank you for this comment. We hope our revisions better connect sand content as	a proxy for the soil characteristics described above (85-89). We agree that moisture	availability/fertility would be more informative metrics than our proxy measure of	soil texture, but given the number of sampling plots and desire to characterize	microsite conditions, soil texture was a more logistically feasible characterization of	soil conditions.*

L101: It would be beneficial for readers not familiar with these terms to provide short definitions in parenthesis, with relevant citations. Mesophication and homogenization are both important processes within the context of global change. However, mesophication seems more specific to Northern America compared to other temperate regions, e.g. Europe.

*This feedback was insightful and easily adopted! We hope our parenthetical	definitions make these concepts more interpretable for an audience not familiar with our	ecoregion (108-111).*
L107-108: What is the reasoning behind the expectation that more sandy soil positively affect shrub richness? Because of less competitive environment? On more loamy soil, fertility can facilitate the formation of dense but less diverse shrub layer (competitive exclusion)? Please provide some ecological background.

*Thank you for pointing out the need for additional context. We hope we provide more	context for why shrub cover and richness are hypothesized to be higher in sandier	substrates (85-91). This also relates to our descriptions of nutrient/water availability,	making for a more cohesive introduction.*

L112: The Introduction section finishes with methodological description, which feels a bit odd. I would strongly recommend omitting this part and rather write some catchy research hypotheses or questions stemming from ecological theory related to plant community assembly. Or at least provide clear aims of the study.

*Thank you for this. We hope the end of our introduction better reflects our working hypotheses (114-121).*

L160: As a sensitivity analysis, I recommend using negative binomial distribution because it handles overdispersion issue, common in count data

*Thank you for this. We found the Poisson distribution for plot richness met diagnostic criteria, while negative binomial distribution was appropriate for abundance models.*

L222-224: Such sentences are more suitable for Discussion. Please only report results (if stat. significant).

*Thank you for this. We hope our updates results are more concise.*

L260-261: Where are hypotheses listed? They seem to be rather scattered throughout the Introduction which might be confusing.

*We edited our Introduction to make our hypotheses more obvious and easier to	reference (116-121)*
L340-341: I would be more specific here. Please expand a bit which species responded more to soil texture, a core soil parameter used in this study*.*

*We hope our example taxa (256-257, 286,,303,307,361-362; Fig 5) and clearer prose clarify this take way in our conclusion, as well as in other parts of our results and discussion.*
