# Supplementary material for: Light availability, not soil texture, correlates with shrub community structure in mesic forest gaps
Source: PLoS One. 2026 Oct 29;21(10):e0361488. doi: 10.1371/journal.pone.0361488 (PMC13718914; doi:10.1371/journal.pone.0361488)
Supplement: Supplementary file 8 [file pone.0361488.s014.docx]

**Response to Reviewer Comments**

Each comment received a direct response in italics and blue font, including updated line numbers for ease of viewing. Thank you for your time and constructive feedback.

Reviewer #2: Geographic location is still missing in the abstract, could be very brief and broad (e.g. eastern N. America)

*Line 34: An easy fix. Thank you.*

What forest types (i.e. which tree species dominate the plots) were studied also seems reasonable to be included in the abstract

*L53-54: We believe this context is more appropriate in the introduction.*

First part of the abstract is still too general, I suggest shortening it or more directly connect to specific findings

*L26-35: We hope our revised abstract is more direct.*

L54-55: what about wind, is it an important disturbance agent in these forests?

*L57-58: This sentence was meant to highlight why fire and drought are not dominant parts of the disturbance regime in relation to the preceding sentence as opposed to be an exhaustive list of factors impacting canopy structure. However, we now explicitly reference storm/wind damage in the following sentence describing gap-phase succession.*

L88-91: these two sentences are very similar in their message, omit one

*L89-91: Agreed. We omitted the first sentence*

L99-101: I would be more specific here, shrubs can be a strong additional filter for herbaceous plants residing beneath that layer (if present)

*L99-101: We hope our more explicit phrasing for the impacts of light interception are acceptable.*

L109: not clear to me what do you mean by “development”? Succession?

*L110: Sorry for the confusion. We meant to highlight human driven fragmentation via housing development that produce building zones and roads that fragment mesic forests and create additional forest edges.*

L128: check again, something is missing?

*L129: Apologies, the incomplete clause was the result of editing sentence structure.*

L145: Why not use soil depth as a predictor of shrub communities? As previously emphasized, and also what the other reviewer was concerned about, using only two explanatory variables (canopy gap fraction and soil sand content) is very basic and limits the whole study

*L145-146: Soil depth of the A1 horizon was initially sampled to contextualize the choice to analyze soil texture from the top 15cm of the soil profile, not as an independent explanatory variable with a priori hypotheses. Since the GLMER models we used included nested random effects and varying slopes for each species, convergence and overfitting issues become more common as model complexity grows. The inclusion of soil depth led to models failing to converge, and models including additional plot-level variables are not likely to improve model fit (Table S6).*

L192: why not using quadratic term for sand content as well?

*L191-194: We did not include a quadratic term for sand because we lacked an* a priori *hypothesis that abundance would exhibit a unimodal response along the observed sand gradient. Exploratory analyses also did not indicate substantial nonlinearity in the relationship between dependent variables and sand similar to those seen for gap fraction (Figs. 6,S3,S4,S5). We opted for a more parsimonious linear specification. We additionally explored models including a quadratic sand term, but these models exhibited convergence and parameter-identifiability issues, suggesting that the available data did not strongly support estimation of a quadratic sand effect.*

L196-204: this whole paragraph is difficult to understand, please try to make it more readable / easier to understand for broader audience

*L197-204: This paragraph was meant to meet minimum requirements for reproducibility of the Bayesian modelling we describe in the preceding paragraph, but we trimmed technical details and used more approachable vocabulary.*

Discussion section should be revised, see my following comments
L324: but your study does not include large gaps covering several hectares, so I suggest to rephrase this part
L325-331: too general and less related to your main findings

*L327-337: This paragraph focuses on additional facets of canopy structure likely to impact understory structure beyond gap fraction. Discussion of larger gaps were omitted to tighten prose, and existing discussion of the importance of gap age was re-enforced with additional citations that directly connect to our findings.*

L332-350: too speculative

*L338-354: Several aspects of our analyses support the importance of unquantified site-level variation impacting shrub community structure, warranting discussion and connection to existing literature. This paragraph is meant to acknowledge our results and provide reasonable site-level factors that could be incorporated into future studies of mesic forest understories. Our most notable changes include shortening our discussion of herbivory and included more robust citations across site-level factors to emphasize empirical rationale for including this paragraph in our MS.*

L351-358: feels a bit off the topic

*L 355-366: This paragraph provides broader context for the low explanatory power of our findings. Predicting metrics like richness or composition is difficult, especially as the local scale of our analyses. We hope edits highlight current limitations of methods in predictive ecology, while also connecting more directly to previous sections of the discussion.*
