# Supplementary material for: Light availability, not soil texture, correlates with shrub community structure in mesic forest gaps
Source: PLoS One. 2026 Oct 29;21(10):e0361488. doi: 10.1371/journal.pone.0361488 (PMC13718914; doi:10.1371/journal.pone.0361488)
Supplement: Peer review history

# Peer review: Decision Letter 0

**DOI:** 10.1371/journal.pone.0361488.r001

**Authors:** Francesco Boscutti

4 May 2026

Dear Dr. Anderson,

Thank you for submitting your manuscript to PLOS ONE. After careful consideration, we feel that it has merit but does not fully meet PLOS ONE’s publication criteria as it currently stands. Therefore, we invite you to submit a revised version of the manuscript that addresses the points raised during the review process.

The reviewers acknowledge the value and potential of your manuscript, as well as the soundness of the statistical approach. However, they also raise concerns that the current interpretation may be too strong relative to the limitations of the field sampling design and the available environmental data.

I therefore invite you to revise the manuscript by strengthening the framing of the study, clarifying the methodological details, acknowledging the limitations more explicitly, and adopting a more cautious and descriptive interpretation of the results.

In particular, please ensure that the revised version avoids causal conclusions that are not fully supported by the dataset and more clearly presents the study as an exploratory contribution to understanding shrub communities in mesic temperate forests.

Please submit your revised manuscript by Jun 18 2026 11:59PM. If you will need more time than this to complete your revisions, please reply to this message or contact the journal office at plosone@plos.org. When you're ready to submit your revision, log on to https://www.editorialmanager.com/pone/ and select the 'Submissions Needing Revision' folder to locate your manuscript file.

- A letter that responds to each point raised by the academic editor and reviewer(s). You should upload this letter as a separate file labeled 'Response to Reviewers'.
- A marked-up copy of your manuscript that highlights changes made to the original version. You should upload this as a separate file labeled 'Revised Manuscript with Track Changes'.
- An unmarked version of your revised paper without tracked changes. You should upload this as a separate file labeled 'Manuscript'.

If you would like to make changes to your financial disclosure, please include your updated statement in your cover letter. Guidelines for resubmitting your figure files are available below the reviewer comments at the end of this letter.

If applicable, we recommend that you deposit your laboratory protocols in protocols.io to enhance the reproducibility of your results. Protocols.io assigns your protocol its own identifier (DOI) so that it can be cited independently in the future. For instructions see: https://journals.plos.org/plosone/s/submission-guidelines#loc-laboratory-protocols. Additionally, PLOS ONE offers an option for publishing peer-reviewed Lab Protocol articles, which describe protocols hosted on protocols.io. Read more information on sharing protocols at https://plos.org/protocols?utm_medium=editorial-email&utm_source=authorletters&utm_campaign=protocols.

As the corresponding author, your ORCID iD is verified in the submission system and will appear in the published article. PLOS supports the use of ORCID, and we encourage all coauthors to register for an ORCID iD and use it as well. Please encourage your coauthors to verify their ORCID iD within the submission system before final acceptance, as unverified ORCID iDs will not appear in the published article. *Only*  the individual author can complete the verification step; PLOS staff *cannot*  verify ORCID iDs on behalf of authors.

We look forward to receiving your revised manuscript.

Kind regards,

Francesco Boscutti

Academic Editor

PLOS One

Journal Requirements:

When submitting your revision, we need you to address these additional requirements.

1. Please ensure that your manuscript meets PLOS ONE's style requirements, including those for file naming. The PLOS ONE style templates can be found at

https://journals.plos.org/plosone/s/file?id=wjVg/PLOSOne_formatting_sample_main_body.pdf and

https://journals.plos.org/plosone/s/file?id=ba62/PLOSOne_formatting_sample_title_authors_affiliations.pdf

2. In your Methods section, please provide additional information regarding the permits you obtained for the work. Please ensure you have included the full name of the authority that approved the field site access and, if no permits were required, a brief statement explaining why.

3. Thank you for stating the following in the Acknowledgments Section of your manuscript:

“This material is based upon work Supported by the National Science Foundation Graduate Research Fellowship Program under Grant No. (2137424). Any opinions, findings, and conclusions or recommendations expressed in this material are those of the author(s) and do not necessarily reflect the views of the National Science Foundation. Support was also provided through the Botanical Society of America’s Bill Dahl Graduate Student Research Award and the Tulipa et Paeonia award from the UW-Madison Botany Department.”

We note that you have provided funding information that is not currently declared in your Funding Statement. However, funding information should not appear in the Acknowledgments section or other areas of your manuscript. We will only publish funding information present in the Funding Statement section of the online submission form.

Please remove any funding-related text from the manuscript and let us know how you would like to update your Funding Statement. Currently, your Funding Statement reads as follows:

“SWA was supported by the National Science Foundation Graduate Research Fellowship Program under Grant No. (2137424). Any opinions, findings, and conclusions or recommendations expressed in this material are those of the author(s) and do not necessarily reflect the views of the National Science Foundation. Support was also provided through the Botanical Society of America’s Bill Dahl Graduate Student Research Award and the Tulipa et Paeonia award from the UW-Madison Botany Department. Funders did not play any role in the study design, data collection and analysis, decision to publish, or preparation of the manuscript

Funder URLs in the order they are mentioned https://www.nsfgrfp.org/

https://botany.org/home/awards/awards-for-students/bsagraduatestudentresearchawards.html

https://botany.wisc.edu/financial-support/”

Please include your amended statements within your cover letter; we will change the online submission form on your behalf.

4. We note that the grant information you provided in the ‘Funding Information’ and ‘Financial Disclosure’ sections do not match.

When you resubmit, please ensure that you provide the correct grant numbers for the awards you received for your study in the ‘Funding Information’ section.

5. We note that Figure S1 Fig1 in your submission contain satellite images which may be copyrighted. All PLOS content is published under the Creative Commons Attribution License (CC BY 4.0), which means that the manuscript, images, and Supporting Information files will be freely available online, and any third party is permitted to access, download, copy, distribute, and use these materials in any way, even commercially, with proper attribution. For these reasons, we cannot publish previously copyrighted maps or satellite images created using proprietary data, such as Google software (Google Maps, Street View, and Earth). For more information, see our copyright guidelines: http://journals.plos.org/plosone/s/licenses-and-copyright.

We require you to either (1) present written permission from the copyright holder to publish these figures specifically under the CC BY 4.0 license, or (2) remove the figures from your submission:

1. You may seek permission from the original copyright holder of Figure(s) [#] to publish the content specifically under the CC BY 4.0 license.

We recommend that you contact the original copyright holder with the Content Permission Form (http://journals.plos.org/plosone/s/file?id=7c09/content-permission-form.pdf) and the following text:

“I request permission for the open-access journal PLOS ONE to publish XXX under the Creative Commons Attribution License (CCAL) CC BY 4.0 (http://creativecommons.org/licenses/by/4.0/). Please be aware that this license allows unrestricted use and distribution, even commercially, by third parties. Please reply and provide explicit written permission to publish XXX under a CC BY license and complete the attached form.”

Please upload the completed Content Permission Form or other proof of granted permissions as an "Other" file with your submission.

In the figure caption of the copyrighted figure, please include the following text: “Reprinted from [ref] under a CC BY license, with permission from [name of publisher], original copyright [original copyright year].”

2. If you are unable to obtain permission from the original copyright holder to publish these figures under the CC BY 4.0 license or if the copyright holder’s requirements are incompatible with the CC BY 4.0 license, please either i) remove the figure or ii) supply a replacement figure that complies with the CC BY 4.0 license. Please check copyright information on all replacement figures and update the figure caption with source information. If applicable, please specify in the figure caption text when a figure is similar but not identical to the original image and is therefore for illustrative purposes only.

The following resources for replacing copyrighted map figures may be helpful:

USGS National Map Viewer (public domain): http://viewer.nationalmap.gov/viewer/

The Gateway to Astronaut Photography of Earth (public domain): http://eol.jsc.nasa.gov/sseop/clickmap/

Maps at the CIA (public domain): https://www.cia.gov/library/publications/the-world-factbook/index.html and https://www.cia.gov/library/publications/cia-maps-publications/index.html

NASA Earth Observatory (public domain): http://earthobservatory.nasa.gov/

Landsat: http://landsat.visibleearth.nasa.gov/

USGS EROS (Earth Resources Observatory and Science (EROS) Center) (public domain): http://eros.usgs.gov/#

Natural Earth (public domain): http://www.naturalearthdata.com/

6. If the reviewer comments include a recommendation to cite specific previously published works, please review and evaluate these publications to determine whether they are relevant and should be cited. There is no requirement to cite these works unless the editor has indicated otherwise.

[Note: HTML markup is below. Please do not edit.]

Reviewers' comments:

Reviewer's Responses to Questions

**Comments to the Author**

1. Is the manuscript technically sound, and do the data support the conclusions?

Reviewer #1: Partly

Reviewer #2: Yes

**********

2. Has the statistical analysis been performed appropriately and rigorously?

Reviewer #1: Yes

Reviewer #2: Yes

**********

3. Have the authors made all data underlying the findings in their manuscript fully available?

The PLOS Data policy

Reviewer #1: No

Reviewer #2: Yes

**********

4. Is the manuscript presented in an intelligible fashion and written in standard English?

Reviewer #1: Yes

Reviewer #2: Yes

**********

Reviewer #1: Dear Authors, dear Editorial Office,

Thank you for the opportunity to review this manuscript, which sheds light on an often overlooked, and “shady” topic. I believe the research idea itself has considerable potential, and the study provides, in general, suitable data to address the two main hypotheses. However, the applied field methodology substantially constrains the extent to which these questions can be meaningfully explored.

Although the sampling sites are distributed across a relatively large area and include a north–south comparison, the overall sample size and survey effort appear limited relative to the likely high environmental heterogeneity. Key contextual information is lacking, such as clear characterization of forest types, more detailed description of canopy gaps beyond light availability, and a more comprehensive approach to soil sampling. As a result, it is difficult to account for substantial environmental “noise” across sites.

Given these limitations, I am skeptical that causal conclusions can be drawn from the current dataset. While it may still be informative to report that shrub species richness tends to be higher in sandier and more open (light-rich) environments compared to more closed and loamy conditions, the current evidence does not appear sufficient to support stronger interpretations.

A further concern is the absence of vegetation cover data, which is a standard and important component of understory vegetation studies, particularly when sampling intensity is relatively low. While presence–absence data have a long tradition in vegetation ecology, and stem counts can provide additional information, these metrics alone may not be sufficient for the stated research aims.

On the positive side, the statistical approaches are well executed and sophisticated, and it would be highly interesting to see how such models perform on a dataset with more detailed vegetation structure and environmental data.

Overall, the combination of limited sample size, high environmental heterogeneity, and restricted response variables (presence–absence and stem counts rather than cover and structural metrics) likely reduces statistical power and limits ecological inference. This makes it difficult to link environmental drivers to shrub diversity patterns.

My recommendation is that the manuscript would benefit from adopting a more exploratory and descriptive framing, as the current design does not appear to support causal interpretations. Although the statistical approaches are well executed and methodologically sound, this manuscript may be appropriate for Q2.

Abstract

The paragraph is generally well written; however, it places too much emphasis on general background rather than the research itself. Approximately half of the abstract reads like an introduction, whereas it should focus more directly on the study. A brief contextual lead-in of one or two sentences would be sufficient, followed by a clearer statement of the research purpose. Greater emphasis should then be placed on the methods, key findings, and their implications.

Introduction

Another major disturbance factor that could be considered is the impact of climate change on ecosystems worldwide. This driver increasingly overrides many natural dynamics, including gap formation and successional patterns. However, this is intended as a suggestion rather than a critical omission.

While the importance of light availability is well addressed in the text, it may be helpful to briefly explain why soil sand content is also a determining factor. This would make lines 107–108 easier to understand. In general, soil sand content refers to the proportion of particles approximately 0.05–2 mm in size, depending on the classification system used. In many ecosystems with highly sandy soils, however, its role can be more complex or even controversial, as rapid water percolation limits the time available for plant uptake. In such cases, higher sand content may have negative effects on vegetation diversity, including shrubs.

The use of more literary expressions such as “myriads” or “a plethora of” might be reconsidered. Although they can improve readability, they may be less appropriate in a scientific context, where more neutral and precise wording is generally preferred.

The aims of the study, including the hypotheses, should be highlighted in a separate, clearly structured list. Otherwise, the chapter is well written, and the topic and the subsequent research are introduced sufficiently well.

Methods

It is not stated in the main text that 16 forest sites were sampled (this information appears only in Supplementary Fig. 1). This should be clearly highlighted, along with the total number of samples collected. As it stands, the reader must infer these values: assuming 16 forest sites with 20 samples each, this would amount to 320 samples in total. Given that each sample covers 50 m², the total sampled area would be 16,000 m2 (1.6 ha). If this interpretation is correct, then despite the sites being distributed across several forest stands, the total sampled area appears relatively small in relation to the study’s aims. Another issue is that very little information is provided about the canopy gaps themselves. It is unclear whether these are typical small-scale gaps (e.g., on the order of one to two tree lengths) or whether they represent quantified canopy gap fractions. As a result, it is difficult for the reader to form a picture of what these gaps actually looked like in the field. While the data handling of gap metrics is described in detail, a more explicit characterization of the gaps (e.g., their size, structure, and typical appearance) would greatly help the readers. Another aspect that is lacking is a clear introduction to the study site. Key information such as soil characteristics, mean annual temperature and precipitation, potential north–south gradients, and other environmental factors is not provided. In addition, details on forest types and land-use history would be valuable. Without this context, readers – particularly those unfamiliar with the region – are left with only a vague understanding that the study was conducted in a temperate forest. A final issue with the field methods concerns the sampling depth, as soil was collected only from the top 15 cm. While these shrub species can likely reach this depth within one or a few years, the success of individual plants is largely determined by root competition, which typically extends well beyond this layer. Consequently, soil texture was characterized only for the uppermost horizon, raising questions about its ecological relevance. In particular, plant performance may depend strongly on deeper soil layers and the properties of the (full) A and other (particularly B) horizons, which were not considered here.

L124–125: Regarding the choice of survey locations, it would be useful to clarify whether site selection was conducted in a GIS environment (i.e., in a semi-blinded manner) or based on prior visual assessment and subsequent random selection in the field. Both approaches are valid, but this part should be specified.

L127–131: The decision not to record vegetation cover is questionable. Cover is a particularly important metric for the investigated species, especially for clonal taxa (e.g., Rubus, Ribes) or species where multiple stems originate from a single rootstock (e.g., Crataegus). More generally, these species can occupy several square meters even as a single individual. Although abundance is counted as one when stems originate from the same individual, the lack of cover data may introduce substantial noise into the results, particularly when assessing species richness or diversity in a heterogeneous understory. Including cover estimates would likely provide more informative and ecologically meaningful data than presence and abundance.

The statistical analyses are appropriate and perhaps even somewhat overly detailed. However, the inclusion of code in the main text is unnecessary and should be reconsidered. A more important question is whether the collected data (small sample size, and abundance without coverage) are suitable for this type of analysis. Nonetheless, I appreciate the range of statistical methods used by the authors.

Results

Fig. 1 – The image appears to be poorly edited in the version I downloaded, although this may be due to the downloading process and not reflect the original quality. Currently, all points are displayed in the same color (green), with a yellow boundary line. Important cartographic elements such as scale, resolution, and a north arrow are missing. In addition, several points overlap, reducing clarity. This could be improved by separating the northern and southern parts into distinct panels and then combining them into a single composite figure. Including proper scaling and additional reference features (e.g., forest stand boundaries or nearby settlements for orientation) would further enhance interpretability.

Overall, the results section is concise and informative, and the figures are adequate and clearly present the findings. However, the frequent occurrence of non-significant results, high standard deviations, and low explained variance raises questions about whether these patterns may be attributable to the study design itself, particularly the limited sample size and the absence of other vegetation measures. The 16 sites are also likely relatively far apart and situated in presumably different forest types, which may introduce substantial environmental heterogeneity and additional noise that is difficult to account for.

Discussion

The first subchapter relates the study’s findings well to those of similar research. In contrast, the second subchapter contains rather general statements, particularly in lines 302–333, that do not appear to be well supported by the present results. While I agree that the mentioned factors can influence shrub communities alongside several other drivers, this discussion would be more appropriately placed and briefly addressed in the introduction. The inclusion of such broader statements here may reflect the lack of strong results, and I am concerned that this is primarily attributable to the limited sampling effort.

Conclusion

It is again rather general and should focus more specifically on how this research contributes to our understanding of shrub communities in deciduous forest ecosystems within temperate climates.

Reviewer #2: General evaluation of the MS

The study is well-framed around canopy gaps, light availability and soil texture, assumed to be key drivers in mesic forest ecology. It clearly articulates why understory shrubs matter, which helps justify the study. Multi-scale perspective (plot vs. stand level) is also a relatively strong point, although it should be more specified in the Abstract. This study brings some insight into the dynamics and ecological determinants of shrub layer communities in Wisconsin’s mesic forests, where shrubs represent ~60% of the woody flora and shade is presumably the major driver of diversity and community composition. Authors analyzed shrub layer communities with respect to light availability (gap fraction) and soil conditions (sand content in top mineral horizon). They found that light (presence of canopy gaps) is more important, while soil sand appeared to be more species-specific driver. Authors discuss the low explanatory power of used predictors and acknowledge that other abiotic (e.g. heterogeneity in light and soil conditions) or biotic (deer herbivory) factors not accounted for are likely even more important in shaping shrub communities under mesic canopies in hardwood forests. I was a bit disappointed to see that only light and soil sand were included. I wonder why other variables were not inventoried at sampling plots? Some of them could be obtained with quick visual examination, no? It feels like a missed opportunity.

In my opinion, one of the main weaknesses are the Title and the Abstract.

For the Title: It is clear and accurate, but slightly broad and generic. “Microsite specificity” is somewhat vague and could be more explicit. My suggestion would be to make it more specific and outcome-oriented, e.g. something like “Canopy gaps drive shrub diversity and species-specific microsite preferences in mesic forests” or “Light availability, not soil texture, shapes shrub community structure in mesic forest gaps”

For the Abstract: (1) The background is slightly wordy and could be tightened. (2) More importantly, the abstract lacks even minimal detail on how the study was conducted (e.g., sampling design, number of plots, metrics). And (3) The conclusion leans on broad ecological ideas without clearly stating what’s novel. For instance, “Our results support previous hypotheses…” is too broad so I recommend trying something more assertive e.g. “These findings highlight light availability as the primary driver of shrub community assembly in mesic forests, while species-specific responses to microsite conditions reveal fine-scale niche partitioning that is not captured at broader spatial scales.”

Regarding the methodological approach, soil sand content alone is a very limited proxy for soil conditions. Authors should clearly state this limitation in the Discussion section. Shrubs are likely impacted by other soil variables, such as soil moisture, organic matter, nutrient availability (N, P) and/or at least soil pH (usually strong effect on plant occurrence patterns).

Specific comments

L36: What type of diversity, which metric? Please be more specific.

L71: Woody growth also means deeper rooting (compared to herbaceous species), making it less susceptible to summer drought (if they occur in mesic forests).

L75-79: That is solid justification of why shrubs are important to study, yet often overlooked. More focus has been placed on trees (e.g. economic value of a forest) and herbs (main diversity assets in temperate forests).

L87: Here the authors address soil moisture availability and soil fertility (productivity), yet these were not included in their approach. They solely rely on soil texture. I think a stronger explanation why this approach seems reasonable is needed in the Introduction.

L101: It would be beneficial for readers not familiar with these terms to provide short definitions in parenthesis, with relevant citations. Mesophication and homogenization are both important processes within the context of global change. However, mesophication seems more specific to Northern America compared to other temperate regions, e.g. Europe.

L107-108: What is the reasoning behind the expectation that more sandy soil positively affect shrub richness? Because of less competitive environment? On more loamy soil, fertility can facilitate the formation of dense but less diverse shrub layer (competitive exclusion)? Please provide some ecological background.

L112: The Introduction section finishes with methodological description, which feels a bit odd. I would strongly recommend omitting this part and rather write some catchy research hypotheses or questions stemming from ecological theory related to plant community assembly. Or at least provide clear aims of the study.

L160: As a sensitivity analysis, I recommend using negative binomial distribution because it handles overdispersion issue, common in count data

L222-224: Such sentences are more suitable for Discussion. Please only report results (if stat. significant).

L260-261: Where are hypotheses listed? They seem to be rather scattered throughout the Introduction which might be confusing.

L340-341: I would be more specific here. Please expand a bit which species responded more to soil texture, a core soil parameter used in this study.

**********

what does this mean?). If published, this will include your full peer review and any attached files.

If you choose “no”, your identity will remain anonymous but your review may still be made public.

**Do you want your identity to be public for this peer review?**  For information about this choice, including consent withdrawal, please see our Privacy Policy

Reviewer #1: No

Reviewer #2: No

**********

[NOTE: If reviewer comments were submitted as an attachment file, they will be attached to this email and accessible via the submission site. Please log into your account, locate the manuscript record, and check for the action link "View Attachments". If this link does not appear, there are no attachment files.]

To ensure your figures meet our technical requirements, please review our figure guidelines: https://journals.plos.org/plosone/s/figures

You may also use PLOS’s free figure tool, NAAS, to help you prepare publication quality figures: https://journals.plos.org/plosone/s/figures#loc-tools-for-figure-preparation.

NAAS will assess whether your figures meet our technical requirements by comparing each figure against our figure specifications.

# Author comment: Author response to Decision Letter 1

**DOI:** 10.1371/journal.pone.0361488.r002

21 Aug 2026

Thank you for your feedback! We hope our response to your general comments and line-item responses in the Response_to_Reviewers document are satisfactory. Your feedback has made for a much stronger manuscript.

Attachment Submitted filename: Response_to_Reviewers.docx

# Peer review: Decision Letter 1

**DOI:** 10.1371/journal.pone.0361488.r003

**Authors:** Francesco Boscutti

15 Sep 2026

Dear Dr. Anderson,

I would ask the authors to carefully revise the manuscript in line with Reviewer 2’s comments, with particular attention to strengthening the abstract, improving the ecological and statistical justification of the analyses, clarifying unclear sections, and refocusing the Discussion on findings directly supported by the study while reducing overly general or speculative interpretations.

Please submit your revised manuscript by Oct 30 2026 11:59PM. If you will need more time than this to complete your revisions, please reply to this message or contact the journal office at plosone@plos.org. When you're ready to submit your revision, log on to https://www.editorialmanager.com/pone/ and select the 'Submissions Needing Revision' folder to locate your manuscript file.

- A letter that responds to each point raised by the academic editor and reviewer(s). You should upload this letter as a separate file labeled 'Response to Reviewers'.
- A marked-up copy of your manuscript that highlights changes made to the original version. You should upload this as a separate file labeled 'Revised Manuscript with Track Changes'.
- An unmarked version of your revised paper without tracked changes. You should upload this as a separate file labeled 'Manuscript'.

If you would like to make changes to your financial disclosure, please include your updated statement in your cover letter. Guidelines for resubmitting your figure files are available below the reviewer comments at the end of this letter.

If applicable, we recommend that you deposit your laboratory protocols in protocols.io to enhance the reproducibility of your results. Protocols.io assigns your protocol its own identifier (DOI) so that it can be cited independently in the future. For instructions see: https://journals.plos.org/plosone/s/submission-guidelines#loc-laboratory-protocols. Additionally, PLOS One offers an option for publishing peer-reviewed Lab Protocol articles, which describe protocols hosted on protocols.io. Read more information on sharing protocols at https://plos.org/protocols?utm_medium=editorial-email&utm_source=authorletters&utm_campaign=protocols.

As the corresponding author, your ORCID iD is verified in the submission system and will appear in the published article. PLOS supports the use of ORCID, and we encourage all coauthors to register for an ORCID iD and use it as well. Please encourage your coauthors to verify their ORCID iD within the submission system before final acceptance, as unverified ORCID iDs will not appear in the published article. *Only*  the individual author can complete the verification step; PLOS staff *cannot*  verify ORCID iDs on behalf of authors.

We look forward to receiving your revised manuscript.

Kind regards,

Francesco Boscutti

Academic Editor

PLOS One

Journal Requirements:

If the reviewer comments include a recommendation to cite specific previously published works, please review and evaluate these publications to determine whether they are relevant and should be cited. There is no requirement to cite these works unless the editor has indicated otherwise.

Please review your reference list to ensure that it is complete and correct. If you have cited papers that have been retracted, please include the rationale for doing so in the manuscript text, or remove these references and replace them with relevant current references. Any changes to the reference list should be mentioned in the rebuttal letter that accompanies your revised manuscript. If you need to cite a retracted article, indicate the article’s retracted status in the References list and also include a citation and full reference for the retraction notice.

[Note: HTML markup is below. Please do not edit.]

Reviewers' comments:

Reviewer's Responses to Questions

**Comments to the Author**

Reviewer #2: (No Response)

**********

2. Is the manuscript technically sound, and do the data support the conclusions?

Reviewer #2: Yes

**********

3. Has the statistical analysis been performed appropriately and rigorously?

Reviewer #2: Yes

**********

4. Have the authors made all data underlying the findings in their manuscript fully available?

The PLOS Data policy

Reviewer #2: Yes

**********

5. Is the manuscript presented in an intelligible fashion and written in standard English?

Reviewer #2: Yes

**********

Reviewer #2: Geographic location is still missing in the abstract, could be very brief and broad (e.g. eastern N. America)

What forest types (i.e. which tree species dominate the plots) were studied also seems reasonable to be included in the abstract

First part of the abstract is still too general, I suggest shortening it or more directly connect to specific findings

L54-55: what about wind, is it an important disturbance agent in these forests?

L88-91: these two sentences are very similar in their message, omit one

L99-101: I would be more specific here, shrubs can be a strong additional filter for herbaceous plants residing beneath that layer (if present)

L109: not clear to me what do you mean by “development”? Succession?

L128: check again, something is missing?

L145: Why not use soil depth as a predictor of shrub communities? As previously emphasized, and also what the other reviewer was concerned about, using only two explanatory variables (canopy gap fraction and soil sand content) is very basic and limits the whole study

L192: why not using quadratic term for sand content as well?

L196-204: this whole paragraph is difficult to understand, please try to make it more readable / easier to understand for broader audience

Discussion section should be revised, see my following comments

L324: but your study does not include large gaps covering several hectares, so I suggest to rephrase this part

L325-331: too general and less related to your main findings

L332-350: too speculative

L351-358: feels a bit off the topic

**********

what does this mean?). If published, this will include your full peer review and any attached files.

If you choose “no”, your identity will remain anonymous but your review may still be made public.

**Do you want your identity to be public for this peer review?**  For information about this choice, including consent withdrawal, please see our Privacy Policy

Reviewer #2: No

**********

[NOTE: If reviewer comments were submitted as an attachment file, they will be attached to this email and accessible via the submission site. Please log into your account, locate the manuscript record, and check for the action link "View Attachments". If this link does not appear, there are no attachment files.]

To ensure your figures meet our technical requirements, please review our figure guidelines: https://journals.plos.org/plosone/s/figures

You may also use PLOS’s free figure tool, NAAS, to help you prepare publication quality figures: https://journals.plos.org/plosone/s/figures#loc-tools-for-figure-preparation.

NAAS will assess whether your figures meet our technical requirements by comparing each figure against our figure specifications.

# Author comment: Author response to Decision Letter 2

**DOI:** 10.1371/journal.pone.0361488.r004

1 Oct 2026

Response to Reviewer Comments:

Each comment received a direct response, including updated line numbers for ease of viewing. Thank you for your time and constructive feedback.

Reviewer #2: Geographic location is still missing in the abstract, could be very brief and broad (e.g. eastern N. America)

Line 34: An easy fix. Thank you.

What forest types (i.e. which tree species dominate the plots) were studied also seems reasonable to be included in the abstract

L53-54: We believe this context is more appropriate in the introduction.

First part of the abstract is still too general, I suggest shortening it or more directly connect to specific findings

L26-35: We hope our revised abstract is more direct.

L54-55: what about wind, is it an important disturbance agent in these forests?

L57-58: This sentence was meant to highlight why fire and drought are not dominant parts of the disturbance regime in relation to the preceding sentence as opposed to be an exhaustive list of factors impacting canopy structure. However, we now explicitly reference storm/wind damage in the following sentence describing gap-phase succession.

L88-91: these two sentences are very similar in their message, omit one

L89-91: Agreed. We omitted the first sentence

L99-101: I would be more specific here, shrubs can be a strong additional filter for herbaceous plants residing beneath that layer (if present)

L99-101: We hope our more explicit phrasing for the impacts of light interception are acceptable.

L109: not clear to me what do you mean by “development”? Succession?

L110: Sorry for the confusion. We meant to highlight human driven fragmentation via housing development that produce building zones and roads that fragment mesic forests and create additional forest edges.

L128: check again, something is missing?

L129: Apologies, the incomplete clause was the result of editing sentence structure.

L145: Why not use soil depth as a predictor of shrub communities? As previously emphasized, and also what the other reviewer was concerned about, using only two explanatory variables (canopy gap fraction and soil sand content) is very basic and limits the whole study

L145-146: Soil depth of the A1 horizon was initially sampled to contextualize the choice to analyze soil texture from the top 15cm of the soil profile, not as an independent explanatory variable with a priori hypotheses. Since the GLMER models we used included nested random effects and varying slopes for each species, convergence and overfitting issues become more common as model complexity grows. The inclusion of soil depth led to models failing to converge, and models including additional plot-level variables are not likely to improve model fit (Table S6).

L192: why not using quadratic term for sand content as well?

L191-194: We did not include a quadratic term for sand because we lacked an a priori hypothesis that abundance would exhibit a unimodal response along the observed sand gradient. Exploratory analyses also did not indicate substantial nonlinearity in the relationship between dependent variables and sand similar to those seen for gap fraction (Figs. 6,S3,S4,S5). We opted for a more parsimonious linear specification. We additionally explored models including a quadratic sand term, but these models exhibited convergence and parameter-identifiability issues, suggesting that the available data did not strongly support estimation of a quadratic sand effect.

L196-204: this whole paragraph is difficult to understand, please try to make it more readable / easier to understand for broader audience

L197-204: This paragraph was meant to meet minimum requirements for reproducibility of the Bayesian modelling we describe in the preceding paragraph, but we trimmed technical details and used more approachable vocabulary.

Discussion section should be revised, see my following comments

L324: but your study does not include large gaps covering several hectares, so I suggest to rephrase this part

L325-331: too general and less related to your main findings

L327-337: This paragraph focuses on additional facets of canopy structure likely to impact understory structure beyond gap fraction. Discussion of larger gaps were omitted to tighten prose, and existing discussion of the importance of gap age was re-enforced with additional citations that directly connect to our findings.

L332-350: too speculative

L338-354: Several aspects of our analyses support the importance of unquantified site-level variation impacting shrub community structure, warranting discussion and connection to existing literature. This paragraph is meant to acknowledge our results and provide reasonable site-level factors that could be incorporated into future studies of mesic forest understories. Our most notable changes include shortening our discussion of herbivory and included more robust citations across site-level factors to emphasize empirical rationale for including this paragraph in our MS.

L351-358: feels a bit off the topic

L 355-366: This paragraph provides broader context for the low explanatory power of our findings. Predicting metrics like richness or composition is difficult, especially as the local scale of our analyses. We hope edits highlight current limitations of methods in predictive ecology, while also connecting more directly to previous sections of the discussion.

Attachment Submitted filename: Response_to_Reviewers2.docx

# Editor report: Decision Letter 2

**DOI:** 10.1371/journal.pone.0361488.r005

**Authors:** Francesco Boscutti

12 Oct 2026

Light availability, not soil texture, correlates with shrub community structure in mesic forest gaps

PONE-D-26-09522R2

Dear Dr. Anderson,

We’re pleased to inform you that your manuscript has been judged scientifically suitable for publication and will be formally accepted for publication once it meets all outstanding technical requirements.

Within one week, you’ll receive an e-mail detailing the required amendments. When these have been addressed, you’ll receive a formal acceptance letter and your manuscript will be scheduled for publication.

An invoice will be generated when your article is formally accepted. Please note, if your institution has a publishing partnership with PLOS and your article meets the relevant criteria, all or part of your publication costs will be covered. Please make sure your user information is up-to-date by logging into Editorial Manager at Editorial Manager® and clicking the ‘Update My Information' link at the top of the page. For questions related to billing, please contact billing support.

If your institution or institutions have a press office, please notify them about your upcoming paper to help maximize its impact. If they’ll be preparing press materials, please inform our press team as soon as possible -- no later than 48 hours after receiving the formal acceptance. Your manuscript will remain under strict press embargo until 2 pm Eastern Time on the date of publication. For more information, please contact onepress@plos.org.

Kind regards,

Francesco Boscutti

Academic Editor

PLOS One

Additional Editor Comments (optional):

Reviewers' comments:

# Editor report: Acceptance letter

**DOI:** 10.1371/journal.pone.0361488.r006

**Authors:** Francesco Boscutti

PONE-D-26-09522R2

PLOS One

Dear Dr. Anderson,

I'm pleased to inform you that your manuscript has been deemed suitable for publication in PLOS One. Congratulations! Your manuscript is now being handed over to our production team.

At this stage, our production department will prepare your paper for publication. This includes ensuring the following:

* All references, tables, and figures are properly cited

* All relevant supporting information is included in the manuscript submission,

* There are no issues that prevent the paper from being properly typeset

You will receive further instructions from the production team, including instructions on how to review your proof when it is ready. Please keep in mind that we are working through a large volume of accepted articles, so please give us a few days to review your paper and let you know the next and final steps.

Lastly, if your institution or institutions have a press office, please let them know about your upcoming paper now to help maximize its impact. If they'll be preparing press materials, please inform our press team within the next 48 hours. Your manuscript will remain under strict press embargo until 2 pm Eastern Time on the date of publication. For more information, please contact onepress@plos.org.

You will receive an invoice from PLOS for your publication fee after your manuscript has reached the completed accept phase. If you receive an email requesting payment before acceptance or for any other service, this may be a phishing scheme. Learn how to identify phishing emails and protect your accounts at https://explore.plos.org/phishing.

If we can help with anything else, please email us at customercare@plos.org.

Thank you for submitting your work to PLOS One and supporting open access.

Kind regards,

PLOS One Editorial Office Staff

on behalf of

Dr. Francesco Boscutti

Academic Editor

PLOS One
